# Efficacy and safety of Runzao Zhiyang capsule for chronic urticaria: a systematic review and meta-analysis of randomized controlled trials

**DOI:** 10.3389/fphar.2023.1200252

**Published:** 2023-08-24

**Authors:** Jian-Feng Zhang, Ying-Dong Wang, Peng Lin, Jun-Chen Li, Chen-Qi Guo, Jing-Bo Zhai, Yu Zhang

**Affiliations:** ^1^ Graduate School, Tianjin University of Traditional Chinese Medicine, Tianjin, China; ^2^ School of Public Health, Tianjin University of Traditional Chinese Medicine, Tianjin, China; ^3^ Department of Dermatology, Tianjin Academy of Traditional Chinese Medicine Affiliated Hospital, Tianjin, China

**Keywords:** Runzao Zhiyang capsule, chronic urticaria, antihistamines, randomized controlled trials, meta-analysis

## Abstract

**Background:** Chronic urticaria (CU) is a commonly seen skin disorder featured by recurring wheals, with or without angioedema, lasting for at least 6 weeks. Runzao Zhiyang capsule (RZC) has been widely applied to treat patients with CU. This study is aimed at systematically evaluating the efficacy and safety of RZC in treating CU.

**Materials and Methods:** Randomized controlled trials (RCTs) of RZC on treating CU from Chinese and English databases were searched. Data were collected by two independent researchers. The Cochrane Collaboration tool was adopted for evaluating the risk of bias. The meta-analysis was performed with Review Manager 5.3 software. Sensitivity analysis and publication bias assessment were conducted by Stata 14.0 software.

**Results:** Totally 27 studies were included in the analysis, involving 2,703 patients. The pooled results showed that compared with second-generation H1-antihistamines (sgAHs) therapy alone, RZC combined with sgAHs is more effective in improving the total effective rate (RR = 1.32, 95% CI: 1.25 to 1.39, *p* < 0.00001), the quality of life measured by Dermatology Life Quality Index (DLQI) (MD = −2.63, 95% CI: −3.68 to −1.58, *p* < 0.00001) and the serum IFN-γ level (SMD = 3.10, 95% CI: 1.58 to 4.62, *p* < 0.0001), and reducing the recurrence rate (RR = 0.39, 95% CI: 0.27 to 0.55, *p* < 0.00001), the serum total IgE level (SMD = −2.44, 95% CI: −3.51 to −1.38, *p* < 0.00001), the serum IL-4 level (SMD = −2.96, 95% CI: −4.10 to −1.83, *p* < 0.00001), and the incidence of adverse events including dizziness, fatigue, dry mouth, and constipation (RR = 0.53, 95% CI: 0.33 to 0.85, *p* = 0.009; RR = 0.46, 95% CI: 0.26 to 0.84, *p* = 0.01; RR = 0.57, 95% CI: 0.34 to 0.95, *p* = 0.03; RR = 0.24, 95% CI: 0.07 to 0.85, *p* = 0.03).

**Conclusion:** The current evidence indicates that RZC may be an efficient therapeutic regimen in patients with CU. Nevertheless, owing to the suboptimal quality of the included studies, more large-scale, well-designed RCTs are required to verify the obtained findings.

**Systematic Review Registration:** https://www.crd.york.ac.uk/PROSPERO/; Identifier: CRD42022313177.

## 1 Introduction

Urticaria is one of the commonly seen dermatoses, characterized by the sudden development of transient hives (wheals) and/or angioedema (Antia et al., 2018). Based on its duration, it can be subdivided into acute and chronic urticaria. In general, episodes with or without angioedema lasting for over 6 weeks are defined as chronic urticaria (CU) (Zuberbier et al., 2022). CU is characterized by intensely pruritic wheals generally undergoing spontaneous resolution within 24 h, and resolves without residual hyperpigmentation or ecchymoses, which is clinically distinct from the wheals’ persistence and purpura/residual hyperpigmentation of urticarial vasculitis (Marzano et al., 2022). It has been shown that the point prevalence of CU in Asian studies is 1.4% in relative to 0.5% and 0.1% in Europe and Northern America, respectively (Fricke et al., 2020). Due to the itching or physical discomfort during outbreaks of CU, recurrent symptoms and long duration, CU imposes a substantial burden on the patients, their families, public health systems, and the society (Gonçalo et al., 2021).

The pathogenesis of CU has not been completely understood. Some studies have demonstrated that mast cells (MCs) and histamine are crucial mediators in etiopathogenesis (Xue et al., 2023). The standard-dosed, second-generation H1-antihistamines (sgAHs) are the first-line pharmacological treatments in guiding the management of CU (Zuberbier et al., 2022). However, licensed doses of sgAHs provide complete symptom relief in less than half of the patients (Agache et al., 2021; Nochaiwong et al., 2021; Patil et al., 2020). When there is no improvement in the clinical symptoms, the dose is increased up to four-fold as second-line therapy (Zuberbier et al., 2022). Among around 50% of patients with CU, symptoms persist even after receiving increased doses of sgAHs or the combination of different sgAHs (Holm et al., 2018; Nochaiwong et al., 2022; Pereyra-Rodriguez et al., 2020). Omalizumab is the monoclonal anti-IgE antibody and is recommended as an add-on therapy for CU in patients who fail to respond to H1-antihistamine according to the EAACI/GA2LEN/EDF/WAO guidelines issued in 2022 (Rubini et al., 2019). Nevertheless, due to the high cost, it is burdensome for most of CU patients (Xiao et al., 2020; Agache et al., 2021). Other drugs, such as cyclosporine and systemic corticosteroids, can also be applied in cases resistant to H1 antihistamines and omalizumab (He et al., 2021). However, cyclosporine is not recommended as a standard treatment because of its systemic adverse effects, such as gastrointestinal symptoms, headache, nephrotoxicity, and elevated blood pressure (Matsubara et al., 2021; Zuberbier et al., 2022). The adverse effect of corticosteroids, such as infection, also limits their clinical application (Yao et al., 2015).

Traditional Chinese medicine (TCM) can be adopted for treating CU following the guideline for the diagnosis and treatment of urticaria in China (2022 edition) (Centre for Urticaria Research of Chinese Society of Dermatology, 2022). As a well-known traditional Chinese patent medicine, Runzao Zhiyang capsule (RZC) is approved for marketing with an approval number of Z20025030 by National Medical Products Administration, and consists of botanical drugs, including *Reynoutria multiflora* (Thunb.) Moldenke [Polygonaceae; Polygoni multiflori radix], *Rehmannia glutinosa* (Gaertn.) DC. [Orobanchaceae; Rehmanniae radix], *Morus alba* L. [Moraceae; Mori folium], *Sophora flavescens* Aiton [Fabaceae; Sophorae flavescentis radix], *Laportea bulbifera* (Siebold & Zucc.) Wedd. [Urticaceae; Laportea herba]. The details of RZC, including the source, composition, description, extraction procedure, its actions, indications, etc., are showed in Supplementary Material S1, the key active ingredients in RZC are summarized in Supplementary Material S2. Modern pharmacological studies have found that several major compounds of RZC, including catalpol, chlorogenic acid, matrine, and formononetin, play a role in inhibiting mast cells degranulation and reducing histamine release (Inami et al., 2013; Lv and Kang, 2016; Xu and An, 2017; Chiu et al., 2021). RZC has immune regulation and anti-inflammation effects, and is often used to relieve itching symptoms of skin disorders (Zheng et al., 2018; Li et al., 2021). Some clinical trials have demonstrated that RZC in combination with sgAHs can significantly improve the efficacy compared with sgAHs alone without serious adverse events. This indicates that RZC may become a potential treatment option for CU (Dou and Zhang, 2019; Ye et al., 2022).

Based on our knowledge, the relatively small sample size and different outcome assessments exist in the single RCT of RZC in treating CU, which failed to offer a systematic and comprehensive assessment of the clinical application of RZC. Till the present, no high-quality systematic reviews on this topic have been published. This study was conducted to critically estimate the efficacy and safety of RZC for treating CU.

## 2 Materials and methods

This study was performed and reported in line with the Preferred Reporting Items for Systematic Review and Meta-Analysis (PRISMA) Guidelines (Page et al., 2021) (Supplementary Material S3). It has been registered on the PROSPERO platform (CRD42022313177).

### 2.1 Eligibility criteria

#### 2.1.1 Types of studies

Only parallel-group RCTs were involved, whereas quasi-RCTs, in which participants were allocated according to the date of birth, hospital record number, or date of admission were not included.

#### 2.1.2 Types of participants

Patients who had a confirmed diagnosis of CU were included irrespective of age, sex, race, and nationality. The recognized diagnostic criteria of CU need to be reported in the included studies, such as guidelines for the diagnosis and therapy of urticaria in China (Centre for Urticaria Research of Chinese Society of Dermatology, 2019) or the EAACI/GA2LEN/EDF/WAO guideline (Zuberbier et al., 2022).

#### 2.1.3 Types of interventions

##### 2.1.3.1 Experimental interventions

The intervention of experimental group was RZC alone or plus sgAHs. Studies with the other TCM treatment in the experimental group (such as other Chinese patent medicine or decoction, Chinese medicine injections, massage, Tai Chi, Qigong, acupuncture, and moxibustion) were not included. There were no limitations on treatment frequency, dosages, and course of RZC.

##### 2.1.3.2 Comparator interventions

The interventions of control group could be placebo, no treatment, or sgAHs. The specific type of sgAHs (Li, 2021; Phinyo et al., 2021) needs to be reported clearly. We investigated the following comparisons:

RZC alone compared with placebo.
RZC alone compared with no treatment.
RZC alone compared with sgAHs.
RZC plus sgAHs compared with sgAHs alone.
RZC plus sgAHs compared with placebo plus sgAHs.

#### 2.1.4 Types of outcome measures

The primary outcome is the total effective rate calculated based on the reduction of the Clinical Symptom Score (CSS) or Urticaria Activity Score (UAS) (Liu et al., 2018). To be specific, the “effectivity” was defined as a more than 60% reduction of CSS or UAS from the baseline for patients (Xiao et al., 2020). The total effective rate is equal to the number of cases labeled as the “effectivity” divided by the total number of cases in one group.

Secondary outcomes include the Dermatology Life Quality Index (DLQI), recurrence rate, serum total immunoglobulin E (IgE) level, serum Interleukin-4 (IL-4) level, serum Interferon-gamma (IFN-γ) level, and adverse reactions.

### 2.2 Literature search

PubMed, Embase, Cochrane Library, Web of Science, China National Knowledge Infrastructure (CNKI), China Science and Technology Journal Database (VIP), Wanfang Database and the Chinese Biomedical Literature Database (SinoMed) were searched from inception to 12 May, 2023. Due to different search regulations across the databases, a search strategy containing medical subject headings and free text words was used and modified if necessary. The search terms such as urticarial, hive, chronic urticaria, Runzao Zhiyang capsule, traditional Chinese Medicine, Chinese patent medicine were selected. Supplementary Material S4 presents the detailed search strategies. To obtain other potentially eligible trials, the reference lists of the relevant reviews, and some trial registration platforms such as Clinical Trials.gov and the Chinese Clinical Trial Registry were searched manually. In addition, no restriction on publication status or language was required.

### 2.3 Literature selecting and data extraction

All the studies identified from the electronic search were managed by NoteExpress Version 3.0. After excluding the duplicates, two reviewers (J. Zhang and Y. Wang) independently inspected the titles and abstracts to eliminate the irrelevant studies. Then, full-texts of the remaining studies were downloaded and read to identify the potentially eligible studies. The data of included articles, such as the first author, year of publication, age, sex, sample size, interventions, treatment course, follow-up period, and outcome indicators were extracted by two authors (J. Zhang and P. Lin) independently. Any disagreement between the two reviewers was resolved by the third author (J. Zhai).

### 2.4 Quality assessment

Following the Cochrane Handbook for Systematic Reviews of Interventions (Higgins et al., 2022), the risk of bias in the involved trials was evaluated independently by two investigators (J. Zhang and J. Li). Seven domains, namely, random sequence generation, allocation concealment, blinding of participants and personnel, blinding of outcome assessment, incomplete outcome data, selective reporting, and other sources of bias were evaluated. Each of them was classified to be low, unclear, or high risk of bias. The “risk of bias” summary and graph showed the risk of bias assessment. Any disagreement was resolved by the third author (J. Zhai).

### 2.5 Statistical analysis

Revman 5.3 version software was applied to carry out meta-analyses. For dichotomous outcomes, the treatment effect was estimated with risk ratio (RR) and 95% confidence intervals (CIs). For continuous outcomes, mean difference (MD) or standardized mean difference (SMD) with 95% CIs was used. Chi-square test was conducted and I^2^ was estimated to assess the heterogeneity across the studies. If a significant heterogeneity (*p* < 0.1 or I^2^>50%) was found, a random effects model was selected to conduct the meta-analysis. Otherwise, a fixed effects model was built. Subgroup analysis was performed based on the course of RZC or the follow-up period if possible. Sensitivity analysis was conducted by Stata 14.0 software. When over ten studies were contained in a meta-analysis, a funnel plot was adopted for evaluating the potential publication bias. Harbord test was carried out to test the publication bias (Sterne et al., 2011).

## 3 Results

### 3.1 Literature screening

**FIGURE 1:**
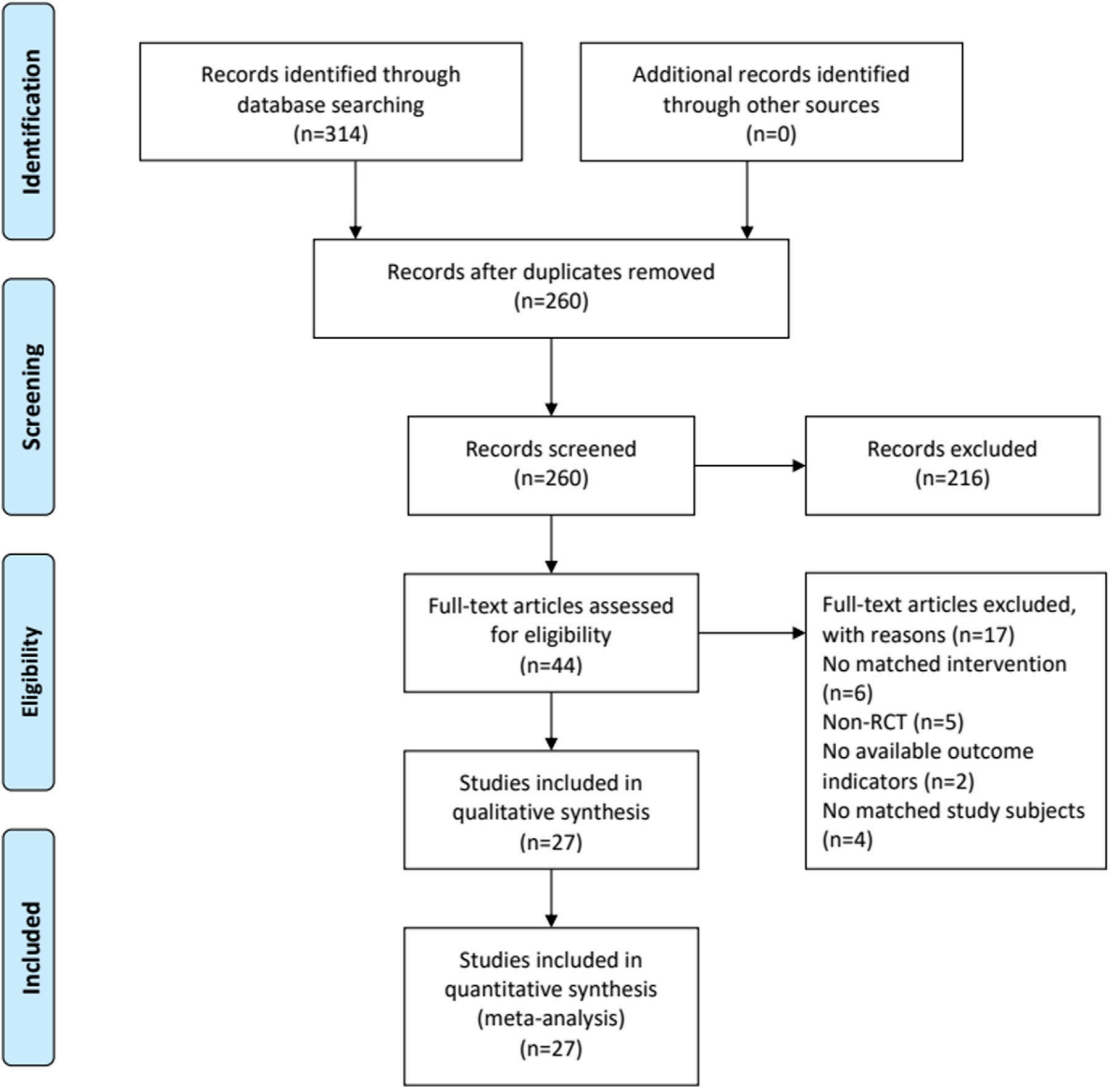
The flowchart of study selection.

Totally 314 potentially relevant articles were initially searched. Fifty-four duplicated studies were excluded by NoteExpress software. Then, 216 articles were removed after screening the titles and abstracts. Among the remaining 44 articles, 17 were further excluded after reading the full-texts. Finally, 27 eligible studies were included (Ai, 2020; Bian et al., 2018; Chen et al., 2016; Chen et al., 2020; Cheng, 2019; Du et al., 2018; Feng et al., 2011; Feng et al., 2016; Huang, 2011; Li Y. et al., 2017; Li, 2019; Liu and Li, 2008; Liu and Yang, 2019; Luo et al., 2016; Lv, 2018; Ma et al., 2014; Sun, 2014; Tan, 2018; Tang and Xu, 2021; Wang and Fang, 2013; Wang et al., 2018; Xiao, 2020; Yang, 2019; Zhang, 2010; Zhang, 2014; Zhang, 2020; Zhou, 2020). Figure 1 displays the flowchart of the study selection.

### 3.2 Study characteristics

All the trials were performed in China. Totally 2,703 patients were enrolled involving 1,368 participants in the RZC plus sgAHs group and 1,335 participants in the sgAHs alone group. They were published from 2008 to 2021. The sample size ranged from 24 to 106. The course of treatment lasted from 2 weeks to 2 months. RZC was taken orally at the dose of 2.0 g three times a day in all the included records. Ten types of sgAHs (desloratadine citrate disodium, cetirizine, desloratadine, levocetirizine, loratadine, ebastine, mizolastine, olopatadine, fexofenadine, and epinastine) were found in the sgAHs alone group. Ten studies reported the duration of follow-up. Only one study (Feng et al., 2011) reported the follow-up period of 7 days, which was the same as the number of studies reporting the follow-up period of 2 months (Bian et al., 2018), 3 months (Feng et al., 2016), and 6–9 months (Zhang, 2014). Six studies (Chen et al., 2016; Liu and Yang, 2019; Luo et al., 2016; Wang, 2018; Wang and Fang, 2013; Li Y. et al., 2017) reported the follow-up periods of 4 weeks to 1 month. Table1 presents the characteristics of the involved studies. The summary of composition characteristics of preparations in all included studies see Supplementary Material S5.

**TABLE 1:** The characteristics of included studies.

First author (publication year)	Sample size (T/C)	Sex (Male/Female)	Age (years)	Course of disease	Interventions (T)	Interventions (C)	Treatment course	Follow-up period	Outcomes
Yang (2019)	61/61	T:30/31	T: 31.8 ± 10.6	T:3m-7y	RZS 2g tid + IC	Desloratadine tablet 5 mg qd	4w	NR	⑦
		C:29/32	C: 30.5 ± 9.8	C:3m-7y					
Chen et al. (2016)	45/35	NR	T/C:15-68	T/C:>6w	RZS 2g tid + IC	Desloratadine tablet 5 mg qd	4w	1m	①④⑦
Feng et al. (2011)	50/50	T:24/26	T:18-62	T:6w-12y	RZS 2g tid + IC	Fexofenadine tablet 60 mg bid	4w	7d	①③⑦
		T:22/28	C:18-65	C:6w-14y					
Lv (2018)	59/59	T:29/30	T:18-68	T:4m-2y	RZS 2g tid + IC	Ebastine tablet 20 mg qd	4w	NR	⑦
		C:33/26	C:18-65	C:3m-2y					
Luo et al. (2016)	63/63	NR	16–65	T:2m-4.2y	RZS 2g tid + IC	Mizolastine tablet 10 mg qd	4w	4w	①③⑦
				C:2m-4.2y					
Tan (2018)	50/50	T:27/23	T:21-60	T:4m-4y	RZS 2g tid + IC	Epinastine capsule 10 mg qd	4w	NR	④⑤⑦
		C:28/22	C:22-59	C:3m-4y					
Xiao (2020)	30/30	T:17/13	T:35.69 ± 2.31	T:2m-4y	RZS 2g tid + IC	Loratadine tablet 5 mg qd	4w	NR	⑦
		C:18/12	C:36.16 ± 2.14	C:3m-5y					
Li (2019)	50/50	T:26/24	T:48.56 ± 3.26	T:2m-4y	RZS 2g tid + IC	Olopatadine tablet 5 mg bid	4w	NR	⑦
		C:25/25	C:49.95 ± 3.86	C:3m-3.5y					
Feng et al. (2016)	60/60	T:32/28	T:21-52	T:7w-2y	RZS 2g tid + IC	Desloratadine citrate disodium capsule 8.8 mg qd	4w	3m	①③④⑦
		C:24/36	C:22-55	C:9w-3y					
Liu and Li (2008)	60/60	T:29/31	T:18-67	T:2m-6y	RZS 2g tid + IC	Loratadine 10 mg qd	4w	NR	①⑦
		C:33/27	C:20-66	C:3m-5y					
Li et al. (2017)	35/35	T:18/17	T:34.23 ± 9.86	T: (25.72 ± 10.41) m	RZS 2g tid + IC	Fexofenadine tablet 60 mg bid	8w	1m	①③⑦
		C:19/16	C:33.12 ± 11.13	C: (26.75 ± 14.50) m					
Tang and Xu (2021)	30/30	T:17/13	T:35.62 ± 3.35	T:3m-6y	RZS 2g tid + IC	Cetirizine 10 mg qd	4w	NR	①②④
		C:18/12	C:34.42 ± 3.39	C:2m-6y					
Bian et al. (2018)	106/94	T:60/46	T: 34.93 ± 2.53	T:3m-6y	RZS 2g tid + IC	Loratadine tablet 1tablet qd	1m	2m	①③④⑤⑦
		C:56/38	C: 35.13 ± 2.15	C:2m-7y					
Du et al. (2018)	42/42	T:21/21	T:32.41 ± 1.76	T:1m-8y	RZS 2g tid + IC	Olopatadine tablet 5 mg bid	2w	NR	①②⑤⑥
		C:23/19	C: 32.54 ± 1.89	C:2m-8y					
Liu and Yang (2019)	50/50	T:28/22	T:16-78	T:2m-2y	RZS 2g tid + IC	Levocetirizine 5 mg qd	2m	4w	①③⑦
		T:31/19	C:17-82	C:2m-2y					
Ai (2020)	24/24	T:14/10	T:35.25 ± 1.39	T:6m-9y	RZS 2g tid + IC	Levocetirizine 10 mg qd	2m	NR	①⑤⑥⑦
		C:13/11	C:35.12 ± 1.46	C:7m-9y					
Zhou (2020)	55/53	T:31/24	T:35.26 ± 1.37	T:5m-6y	RZS 2g tid + IC	Ebastine 10 mg qd	4w	NR	②③⑦
		C:30/23	C:35.19 ± 1.31	C:6m-5.5y					
Ma et al. (2014)	56/54	T:32/24	T:20-65	T:6w-28w	RZS 2g tid + IC	Mizolastine 10 mg qd	4w	NR	①⑦
		C:31/23	C:21-62	C:6w-27w					
Sun (2014)	42/39	T:28/14	T:13-41	T:5m-8y	RZS 2g tid + IC	Desloratadine citrate disodium tablets	2w	NR	①⑦
		C:21/18	C:15-43	C:9m-8y		8.8 mg qn			
Chen et al. (2020)	35/35	NR	T:38.5 ± 3.1	T:2m-2y	RZS 2g tid + IC	Ebastine 10 mg qd	4w	NR	①②④⑤⑥⑦
			C:38.5 ± 3.1	C:2m-2y					
Zhang (2020)	45/45	T:23/22	T:40.25 ± 1.7	T:5m-5y	RZS 2g tid + IC	Levocetirizine 5 mg qd	4w	NR	①
		C:24/21	C:40.15 ± 1.25	C:6m-5y					
Wang and Fang (2013)	46/46	T:30/16	T:35.8 ± 1.5	T:2m-5y	RZS 2g tid + IC	Mizolastine tablet 10 mg qd	4w	1m	①③⑦
		C:28/18	C: 35.7 ± 1.6	C:2m-6y					
Cheng (2019)	50/50	T:28/22	T: 26.21 ± 2.11	T:1y-3y	RZS 2g tid + IC	Epinastine tablet 10 mg qd	4w	NR	⑦
		C:27/23	C: 26.26 ± 2.24	C:1y-3y					
Wang et al. (2018)	64/62	NR	T:>16; C:>16	T:>6w; C:>6w	RZS 2g tid + IC	Ebastine 10 mg qd	4w	4w	①②③⑦
Zhang (2014)	42/42	NR	T:40.62 ± 2.77	T:9m-7y	RZS 2g tid + IC	Ebastine capsule 10 mg qd	1m	6–9 m	②③⑦
			C:40.62 ± 2.77	C:9m-7y					
Huang (2011)	80/80	T:36/44	T:18-55	T:2m-8y	RZS 2g tid + IC	Mizolastine sustained-release tablets 10 mg qd	4w	NR	①⑦
		C:42/38	C:18-55	C:2m-8y					
Zhang (2010)	38/36	NR	T:16-63	T:2m-3y	RZS 2g tid + IC	Mizolastine sustained-release tablets 10 mg qn	2w	NR	①⑦
			C:16-63	C:2m-3y					

Note: T, treatment; C, control; IC, interventions in control group; qd, quaque die; bid, bis in die; tid, ter in die; qn, quaque nocte; d, day; w, week; m, month; ① Total effective rate; ② Dermatology life quality index (DLQI); ③ Recurrence rate; ④ Serum total IgE level; ⑤ Serum IL-4, level; ⑥ Serum IFN-γ, level; ⑦ The incidence of adverse events.

### 3.3 Assessment of risk of bias

**FIGURE 2:**
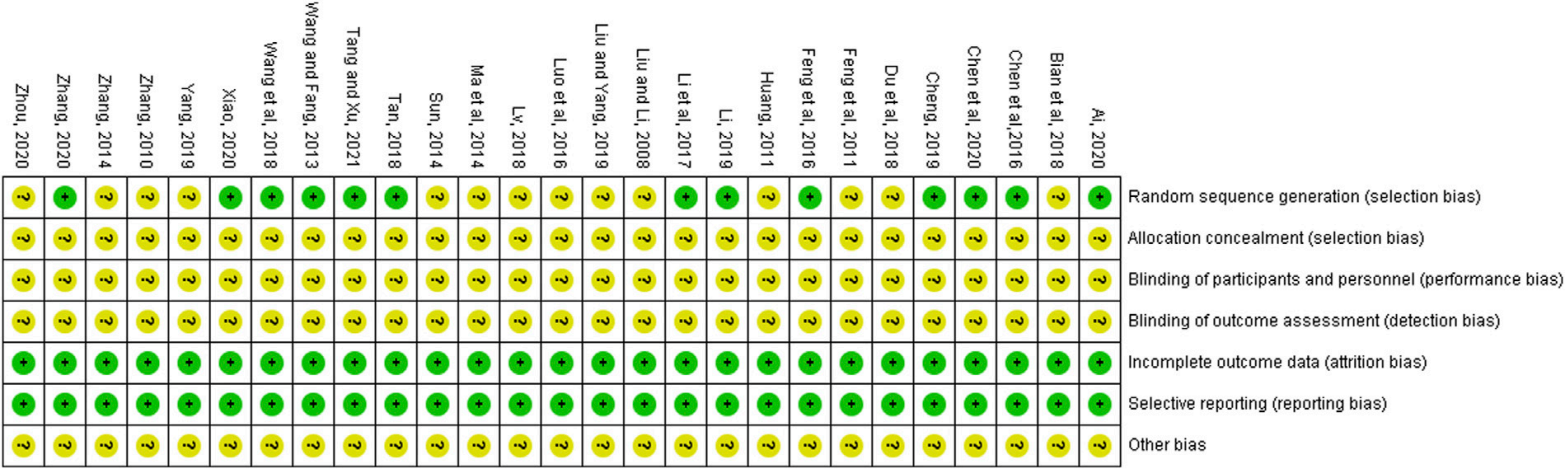
Bias risk assessment of included studies.

All of the 27 RCTs mentioned the random. Among them, 12 trials showed the use of random number tables (Chen et al., 2016; Chen et al., 2020; Cheng, 2019; Feng et al., 2016; Li Y. et al., 2017; Li, 2019; Tan, 2018; Tang and Xu, 2021; Wang and Fang, 2013; Wang et al., 2018; Xiao, 2020; Zhang, 2020), and one trial used the method of random touch balls (Ai, 2020). However, in the remaining studies, the specific random method was not described (Bian et al., 2018; Du et al., 2018; Feng et al., 2011; Huang, 2011; Liu and Li, 2008; Liu and Yang, 2019; Luo et al., 2016; Lv, 2018; Ma et al., 2014; Sun, 2014; Yang, 2019; Zhang, 2010; Zhang, 2014; Zhou, 2020). None of the studies mentioned allocation concealment, blinding of participants and personnel, as well as blinding of outcome assessment. The 27 included articles had complete data and no selectively reported results. Other risk of biases was classified as unclear due to the insufficient information. Figure 2 presents the results of the risk of bias evaluation.

### 3.4 Results of meta-analyses

#### 3.4.1 Primary outcomes

##### 3.4.1.1 Total effective rate

**FIGURE 3:**
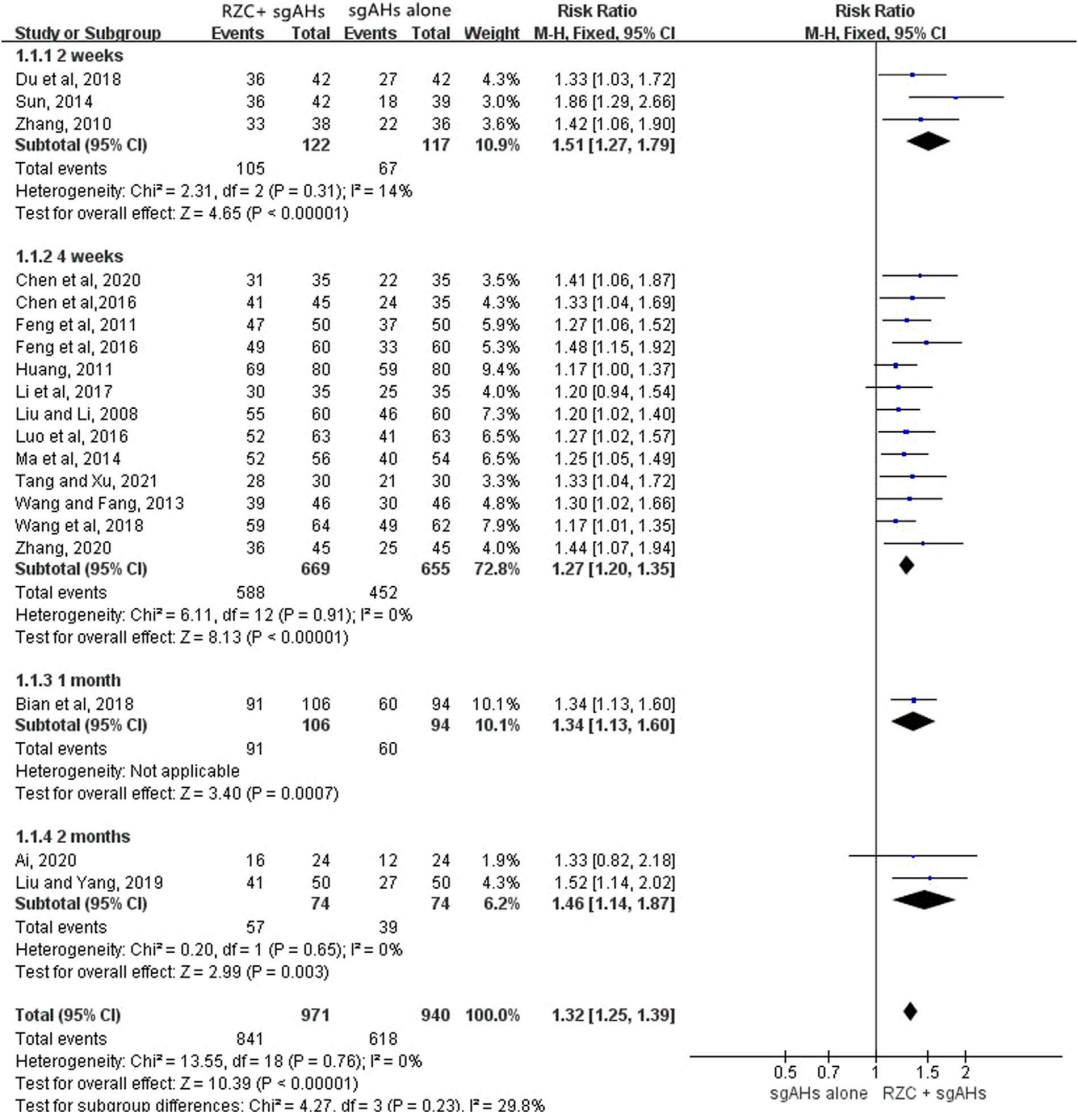
Forest plot for total effective rate.

Nineteen trials involving 1,911 patients (971 in the RZC plus sgAHs group and 940 in the sgAHs alone group) reported the total effective rate (Ai, 2020; Bian et al., 2018; Chen et al., 2016; Chen et al., 2020; Du et al., 2018; Feng et al., 2011; Feng et al., 2016; Huang, 2011; Li Y. et al., 2017; Liu and Li., 2008; Liu and Yang, 2019; Luo et al., 2016; Ma et al., 2014; Sun, 2014; Tang and Xu, 2021; Wang and Fang, 2013; Wang et al., 2018; Zhang, 2010; Zhang, 2020). According to the result of a meta-analysis involving 19 studies, the combination of RZC and sgAHs could obviously improve the total effective rate compared with sgAHs alone (RR = 1.32, 95% CI: 1.25 to 1.39, *p* < 0.00001, Figure 3). A subgroup analysis was performed in accordance with the course of RZC. The total effective rate in the RZC with a course of 2 weeks plus sgAHs group was notably higher than that in the sgAHs alone group (RR = 1.51, 95% CI: 1.27 to 1.79, *p* < 0.00001). The similar findings were obtained after sgAHs treatment plus RZC with a course of 4 weeks (RR = 1.27, 95% CI: 1.20 to 1.35, *p* < 0.00001), 1 month (RR = 1.34, 95% CI: 1.13 to 1.60, *p* = 0.0007), and 2 months (RR = 1.46, 95% CI: 1.14 to 1.87, *p* = 0.003).

#### 3.4.2 Secondary outcomes

##### 3.4.2.1 DLQI

**FIGURE 4:**
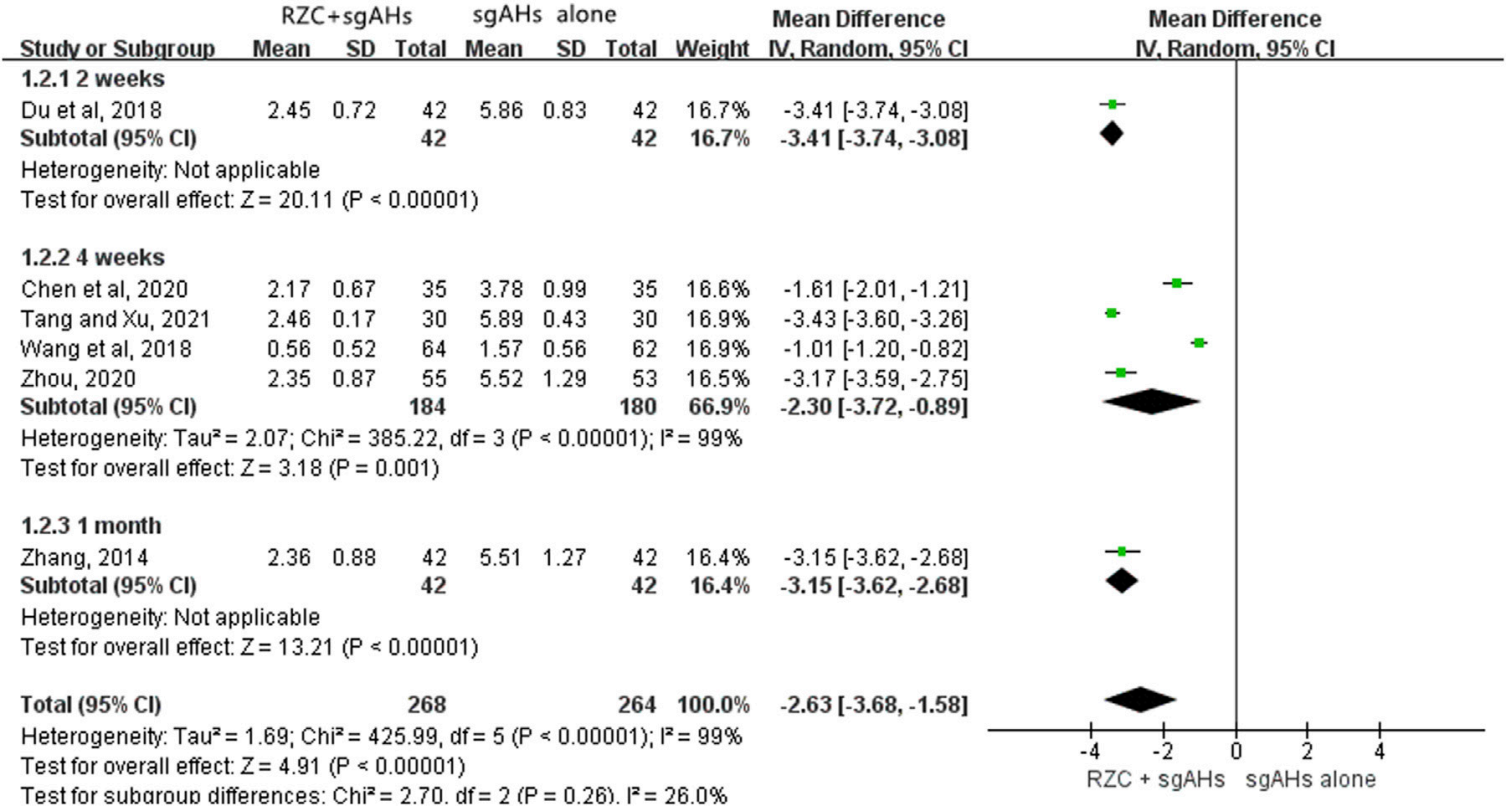
Forest plot for DLQI.

DLQI is one of the vital indicators which can be adopted for assessing the quality of life in the patients with urticaria. Six studies involving 532 participants reported the DLQI (Chen et al., 2020; Du et al., 2018; Tang and Xu, 2021; Wang et al., 2018; Zhang, 2014; Zhou, 2020). The results of a meta-analysis involving six trials suggested that in relative to sgAHs alone, RZC combined with sgAHs significantly improved the quality of life measured by DLQI (MD = −2.63, 95% CI: −3.68 to −1.58, *p* < 0.00001, Figure 4).The results of subgroup analyses showed that RZC combined with sgAHs improved the quality of life measured by DLQI to a great extent compared with sgAHs alone regardless of the treatment course of 2 weeks (MD = −3.41, 95% CI: −3.74 to −3.08, *p* < 0.00001), 4 weeks (MD = −2.30, 95% CI: −3.72 to −0.89, *p* = 0.001), or 1 month (MD = −3.15, 95% CI: −3.62 to −2.68, *p* < 0.00001).

##### 3.4.2.2 Recurrence rate

Ten studies involving 818 participants (447 cases in the RZC combined with sgAHs group and 371 cases in the sgAHs alone group) reported the recurrence rate. One study [Zhou (2020)] reported the number of urticaria recurrence (3 of 55 patients in the RZC plus sgAHs group and 10 of 53 patients in the sgAHs alone group), but did not mention a specific follow-up period, suggesting a lower recurrence rate in the RZC plus sgAHs group relative to the sgAHs alone group (RR = 0.29, 95% CI:0.08 to 0.99, *p* = 0.05).

**FIGURE 5:**
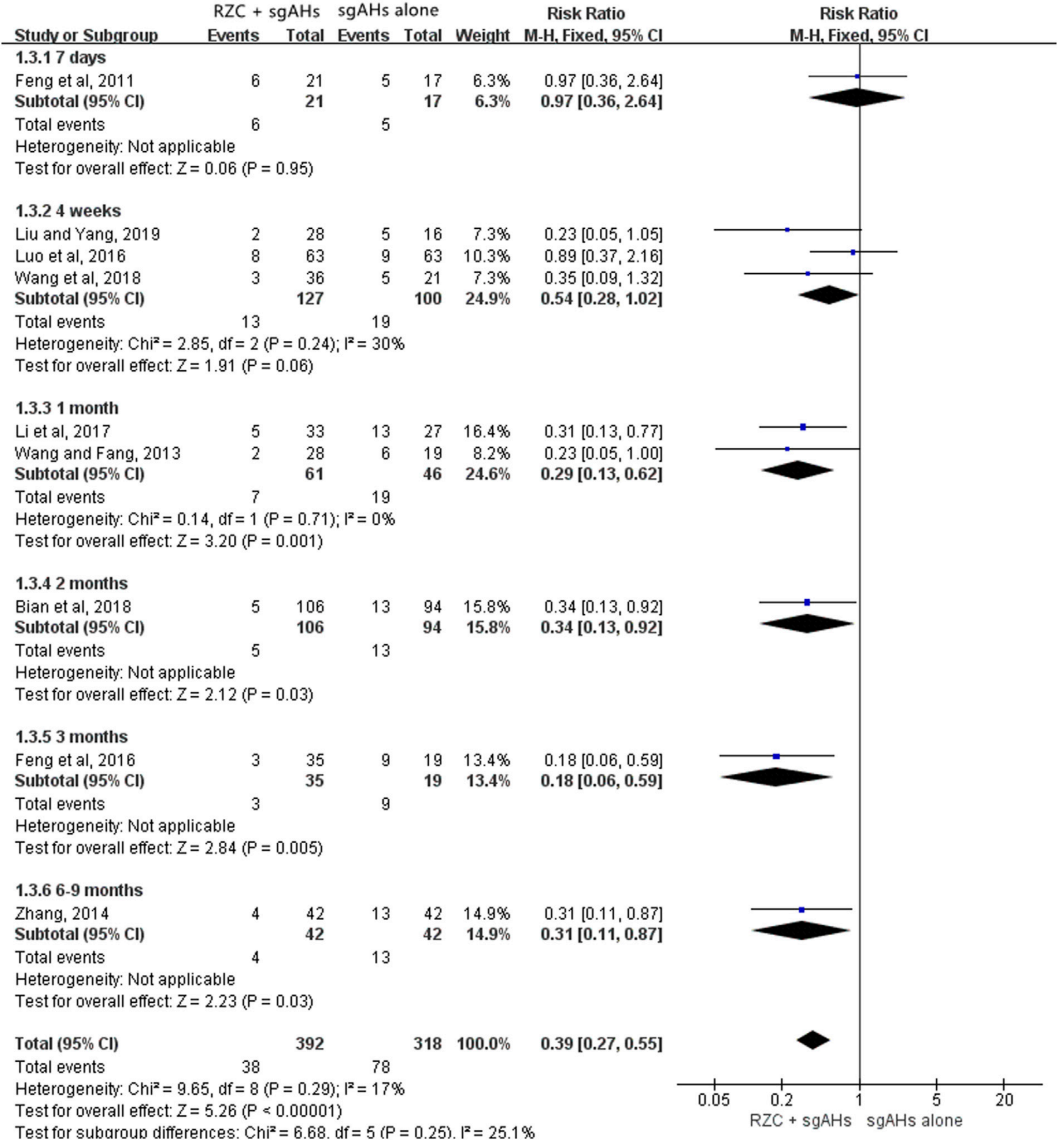
Forest plot for recurrence rate.

A meta-analysis involving the remaining nine studies was conducted (Bian et al., 2018; Feng et al., 2011; Feng et al., 2016; Li Y. et al., 2017; Liu and Yang, 2019; Luo et al., 2016; Wang and Fang, 2013; Wang et al., 2018; Zhang, 2014). A subgroup analysis was carried out based on the follow-up period. The findings of the meta-analysis involving the nine studies demonstrated that the recurrence rate in the RZC plus sgAHs group was lower than that in the sgAHs alone group (RR = 0.39, 95% CI: 0.27 to 0.55, *p* < 0.00001, Figure 5), with the statistical significance. No statistical difference was found after a follow-up period of 7 days (RR = 0.97, 95% CI: 0.36 to 2.64, *p* = 0.95) or 4 weeks (RR = 0.54, 95% CI: 0.28 to 1.02, *p* = 0.06). However, the recurrence rate in the RZC combined with sgAHs group was significantly lower when compared with that in the sgAHs alone group after the follow-up period of 1 month (RR = 0.29, 95% CI: 0.13 to 0.62, *p* = 0.001), 2 months (RR = 0.34, 95% CI: 0.13 to 0.92, *p* = 0.03), 3 months (RR = 0.18, 95% CI: 0.06 to 0.59, *p* = 0.005), and 6–9 months (RR = 0.31, 95% CI: 0.11 to 0.87, *p* = 0.03).

Table 2 shows subgroup analyses on the recurrence rate based on the course of treatment and follow-up period. A lower recurrence rate was found in the RZC with a course of 4 weeks plus sgAHs group in relative to the sgAHs alone group after a follow-up period of 1 month (RR = 0.28, 95% CI: 0.13 to 0.60, *p* = 0.001) or 3 months (RR = 0.25, 95% CI: 0.08 to 0.81, *p* = 0.02). However, the statistical difference in the recurrence rate was not identified between the two groups after a follow-up period of 7 days (RR = 0.97, 95% CI: 0.36 to 2.64, *p* = 0.95) or 4 weeks (RR = 0.67, 95% CI: 0.32 to 1.39, *p* = 0.28). The recurrence rate in the RZC combined with sgAHs group was lower than that in the sgAHs alone group when patients took RZC for 1 month and underwent a follow-up period of 2 months (RR = 0.34, 95% CI: 0.13 to 0.92, *p* = 0.03) or 6–9 months (RR = 0.31, 95% CI: 0.11 to 0.87, *p* = 0.03). Nevertheless, no obvious difference existed in recurrence rate between the two groups after RZC treatment with a course of 2 months, and a follow-up period of 4 weeks (RR = 0.23, 95% CI: 0.05 to 1.05, *p* = 0.06).

**TABLE 2:** Subgroup analysis results of the recurrence rate based on course of RZC and follow-up period.

Included study	Follow-up period	Number of recurrence (TG)	Sample size (TG)	Number of recurrence (CG)	Sample size (CG)	RR	95% CI	*p*-value
Treatment course of 4 weeks								
Feng et al. (2011)	7d	6	21	5	17	0.97 0.36–2.64		0.95
Luo et al., 2016; Wang et al., 2018	4w	11	99	14	84	0.67 0.32–1.39		0.28
Li et al., 2017; Wang and Fang, 2013	1m	7	61	19	46	0.28 0.13–0.60		0.001
Feng et al. (2016)	3m	3	25	9	19	0.25 0.08–0.81		0.02
Treatment course of 1 month								
Bian et al. (2018)	2m	5	106	13	94	0.34 0.13–0.92		0.03
Zhang (2014)	6–9m	4	42	13	42	0.31 0.11–0.87		0.03
Treatment course of 2 months								
Liu and Yang (2019)	4w	2	28	5	16	0.23 0.05–1.05		0.06

TG, treatment group; CG, control group; RR, risk ratio; d, day; w, week; m, month.

##### 3.4.2.3 Serum total IgE level

**FIGURE 6:**
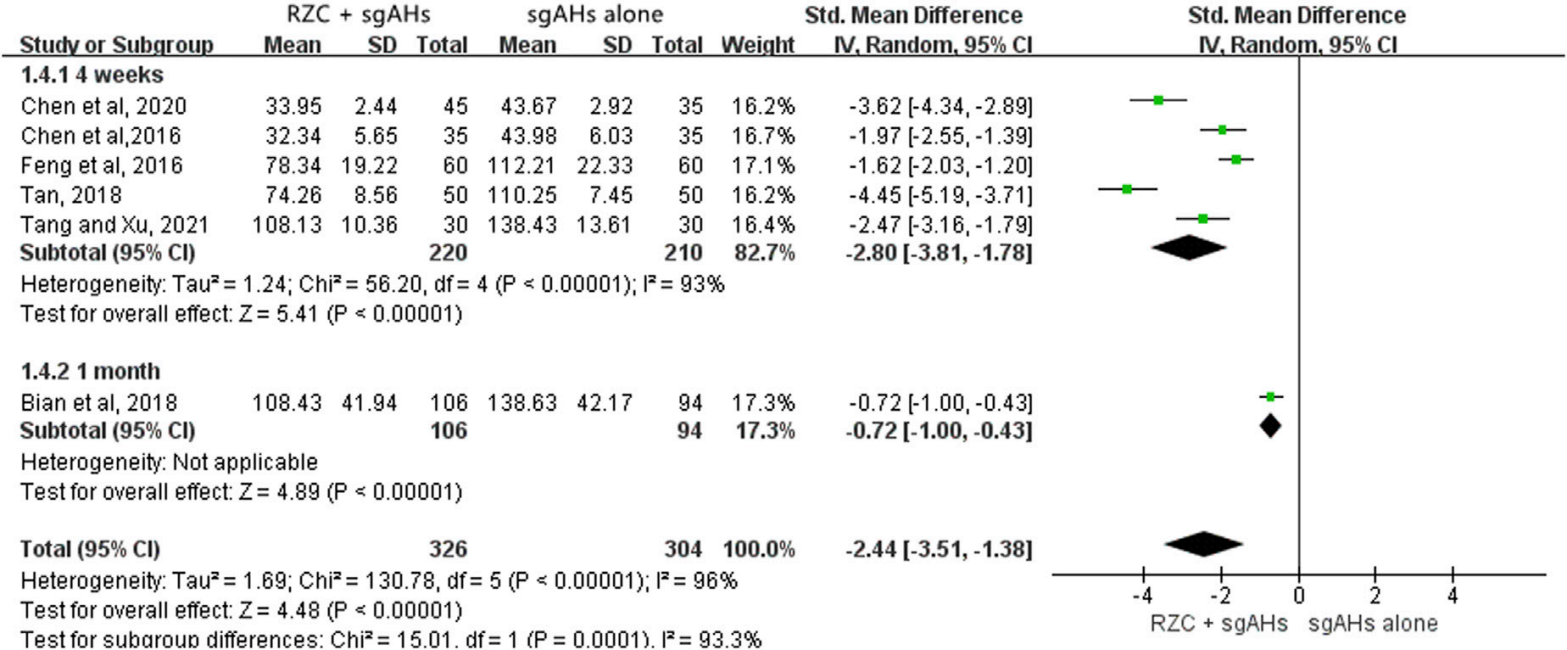
Forest plot for serum total IgE level.

Six studies involving 630 participants reported the serum total IgE level with different units, including iu/mL, ng/mL, and pg/mL (Bian et al., 2018; Chen et al., 2016; Chen et al., 2020; Feng et al., 2016; Tan, 2018; Tang and Xu, 2021). Therefore, SMD was used to describe the effects. The pooled result indicated that the RZC combined with sgAHs group obviously decreased the serum total IgE level in relative to the sgAHs alone group (SMD = −2.44, 95% CI: −3.51 to −1.38, *p* < 0.00001, Figure 6). The findings of subgroup analyses demonstrated that the RZC plus sgAHs group could lower the serum total IgE level more than the sgAHs alone group regardless of the treatment course of 4 weeks (SMD = −2.80, 95% CI: −3.81 to −1.78, *p* < 0.00001) or 1 month (SMD = −0.72, 95% CI: −1.00 to −0.43, *p* < 0.00001).

##### 3.4.2.4 Serum IL-4 level

**FIGURE 7:**
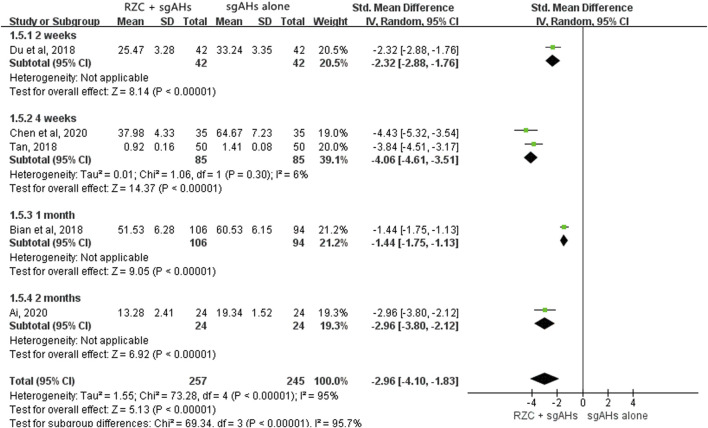
Forest plot for serum IL-4 level.

As five studies presented the serum IL-4 level with different units, such as pg/mL, ng/mL and ng/L, SMD was applied to describe the effects (Ai, 2020; Bian et al., 2018; Chen et al., 2020; Du et al., 2018; Tan, 2018). According to the pooled results, the combination of RZC and sgAHs significantly reduced serum IL-4 level compared with the sgAHs alone group (SMD = −2.96, 95% CI: −4.10 to −1.83, *p* < 0.00001, Figure 7). The results of subgroup analyses based on the treatment course were similar regardless of the treatment course of 2 weeks (SMD = −2.32, 95% CI: −2.88 to −1.76, *p* < 0.00001), 4 weeks (SMD = −4.06, 95% CI: −4.61 to −3.51, *p* < 0.00001), 1 month (SMD = −1.44, 95% CI: −1.75 to −1.13, *p* < 0.00001) or 2 months (SMD = −2.96, 95% CI: −3.80 to −2.12, *p* < 0.00001).

##### 3.4.2.5 Serum IFN-γ level

**FIGURE 8:**
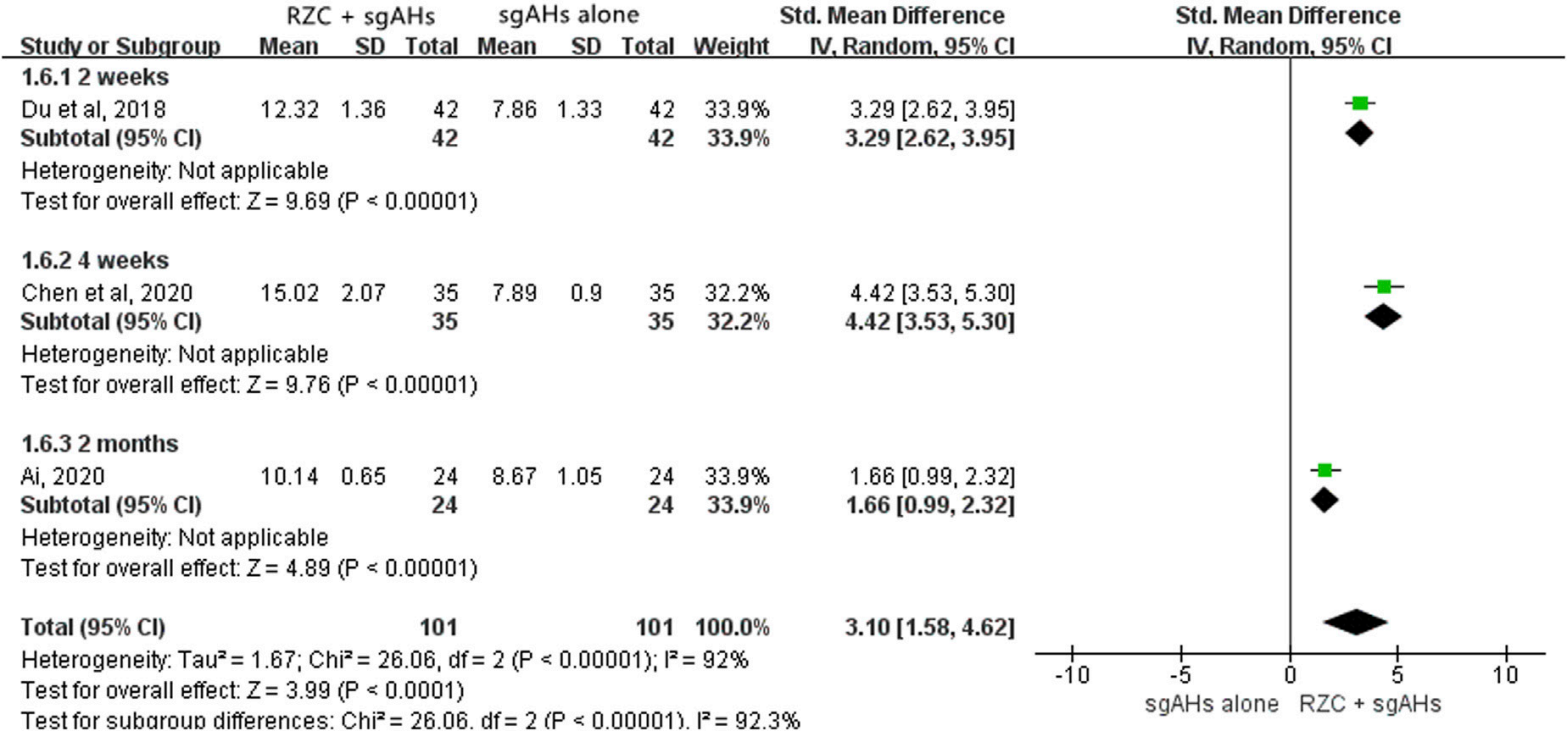
Forest plot for serum IFN-γ level.

Three studies reported the serum IFN-γ level (Ai, 2020; Chen et al., 2020; Du et al., 2018). The unit “pg/mL” was used in the two studies, with “ng/mL” being used in another one. The result of meta-analysis suggested that the serum IFN-γ level in the RZC combined with sgAHs group was statistically higher than that in the sgAHs alone group (SMD = 3.10, 95% CI: 1.58 to 4.62, *p* < 0.0001, Figure 8). The findings of subgroup analyses showed that the RZC plus sgAHs group increased the serum IFN-γ level compared with the sgAHs alone group (SMD = 3.29, 95% CI: 2.62 to 3.95, *p* < 0.00001, a treatment course of 2 weeks, SMD = 4.42, 95% CI: 3.53 to 5.30, *p* < 0.00001, a treatment course of 4 weeks, SMD = 1.66, 95% CI: 0.99 to 2.32, *p* < 0.00001, and a treatment course of 2 months).

##### 3.4.2.6 The incidence of adverse events

A total of 24 studies showed adverse events (Ai, 2020; Bian et al., 2018; Chen et al., 2016; Chen et al., 2020; Cheng, 2019; Feng et al., 2011; Feng et al., 2016; Huang, 2011; Li Y. et al., 2017; Li, 2019; Liu and Li, 2008; Liu and Yang, 2019; Luo et al., 2016; Lv, 2018; Ma et al., 2014; Sun, 2014; Tan, 2018; Wang and Fang, 2013; Wang et al., 2018; Xiao, 2020; Yang, 2019; Zhang, 2010; Zhang, 2014; Zhou, 2020). Only one study (Wang et al., 2018) presented that adverse events occurred in 5 of 64 cases in the RZC plus sgAHs group and 8 of 62 cases in the sgAHs alone group, with no obvious difference in the incidence of adverse events between the two groups (RR = 0.61, 95% CI: 0.21 to 1.75, *p* = 0.35). Nevertheless, the specific symptoms were not reported.

Eleven symptoms on adverse events including drowsiness, dizziness, headaches, fatigue, dry mouth, palpitation, diarrhea, stomach discomfort, constipation, nausea and vomiting and sleepiness were involved in the remaining 23 studies. Subgroup analyses were performed according to different symptoms of adverse events. The pooled results indicated that the RZC combined with sgAHs had a lower incidence of dizziness (RR = 0.53, 95% CI: 0.33 to 0.85, *p* = 0.009), fatigue (RR = 0.46, 95% CI: 0.26 to 0.84, *p* = 0.01), dry mouth (RR = 0.57, 95% CI: 0.34 to 0.95, *p* = 0.03), and constipation (RR = 0.24, 95% CI: 0.07 to 0.85, *p* = 0.03) in relative to the sgAHs alone. Nevertheless, no obvious difference was found in the incidence of drowsiness (RR = 0.75, 95% CI: 0.48 to 1.18, *p* = 0.22), headaches (RR = 0.56, 95% CI: 0.12 to 2.57, *p* = 0.45), palpitation (RR = 0.14, 95% CI: 0.01 to 2.70, *p* = 0.19), diarrhea (RR = 2.60, 95% CI: 0.61 to 11.03, *p* = 0.19), stomach discomfort (RR = 1.52, 95% CI: 0.67 to 3.47, *p* = 0.32), nausea and vomiting (RR = 0.50, 95% CI: 0.09 to 2.69, *p* = 0.42), and sleepiness (RR = 0.50, 95% CI: 0.05 to 5.37, *p* = 0.57) between the two groups. The details are presented in Table 3.

**TABLE 3:** The results of the subgroup analysis of the incidence of adverse events.

Adverse event Symptoms	Number of Studies	Heterogeneity test		Effect of the model	Pooled results		*p*-value
		*p*-value	I^2^ (%)		RR	95% CI	
Drowsiness	17	0.93	0	Fixed	0.75	0.48–1.18	0.22
Dizziness	17	0.98	0	Fixed	0.53	0.33–0.85	0.009
Headaches	2	0.16	48	Fixed	0.56	0.12–2.57	0.45
Fatigue	11	0.79	0	Fixed	0.46	0.26–0.84	0.01
Dry mouth	14	0.71	0	Fixed	0.57	0.34–0.95	0.03
Palpitation	1	—	—	Fixed	0.14	0.01–2.70	0.19
Diarrhea	3	0.33	10	Fixed	2.60	0.61–11.03	0.19
Stomach discomfort	8	0.84	0	Fixed	1.52	0.67–3.47	0.32
Constipation	5	0.95	0	Fixed	0.24	0.07–0.85	0.03
Nausea and vomiting	3	0.83	0	Fixed	0.50	0.09–2.69	0.42
Sleepiness	1	—	—	Fixed	0.50	0.05–5.37	0.57

#### 3.4.3 Additional analysis

##### 3.4.3.1 Sensitivity analysis

**FIGURE 9:**
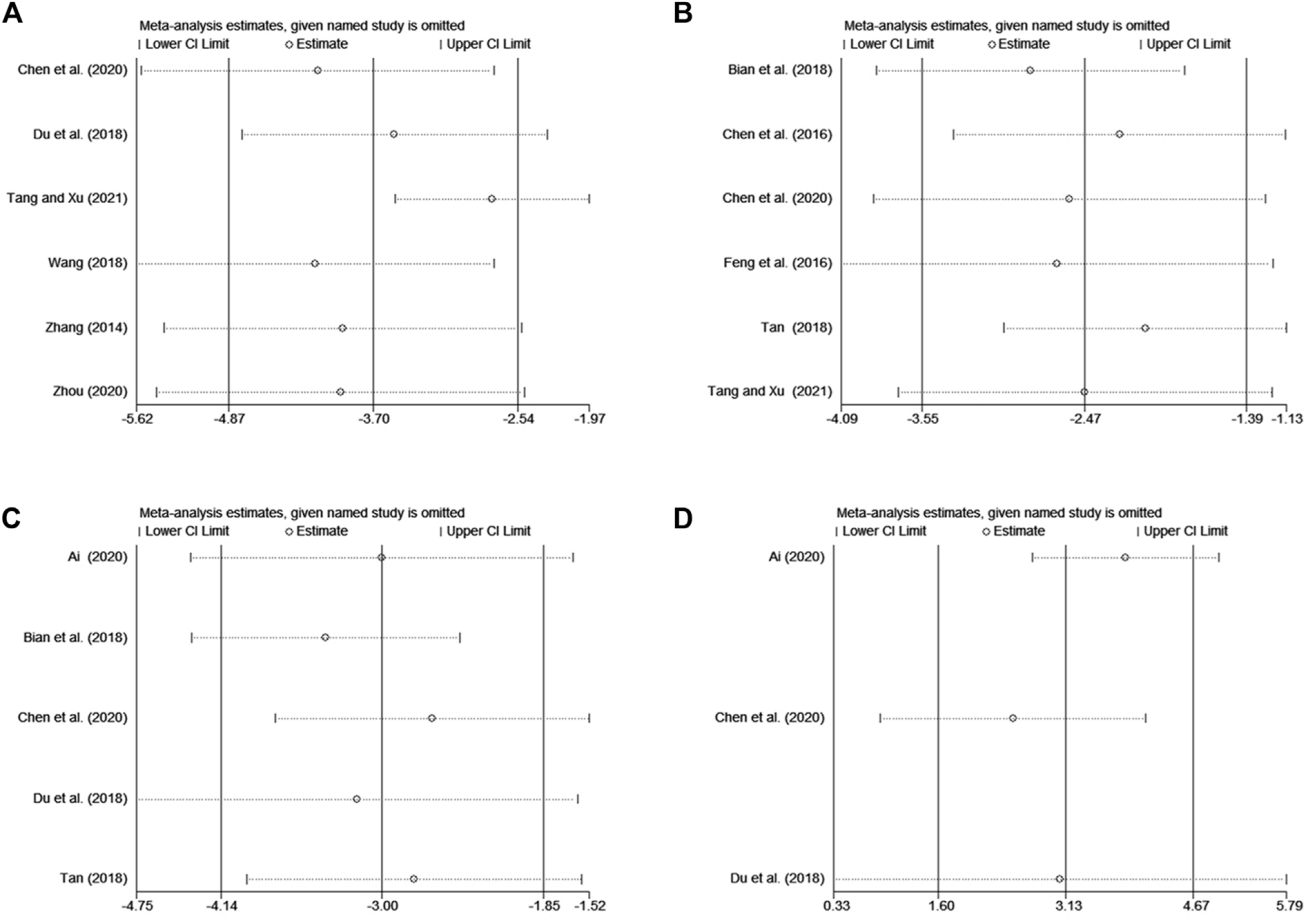
The results of sensitivity analysis. **(A)** DLQI, **(B)** serum total IgE level, **(C)** serum IL-4 level, **(D)** serum IFN-γ level.

The sensitivity analyses were performed using the method of omitting individual studies one by one to evaluate the influence on pooled results due to the heterogeneity of the meta-analyses on the DLQI, serum total IgE level, serum IL-4 level, and serum IFN-γ level. Then, it was demonstrated that omitting individual trials for the above outcome indicator made no difference to the overall results, suggesting that the pooled results were robust (Figure 9).

##### 3.4.3.2 Publication bias

**FIGURE 10:**
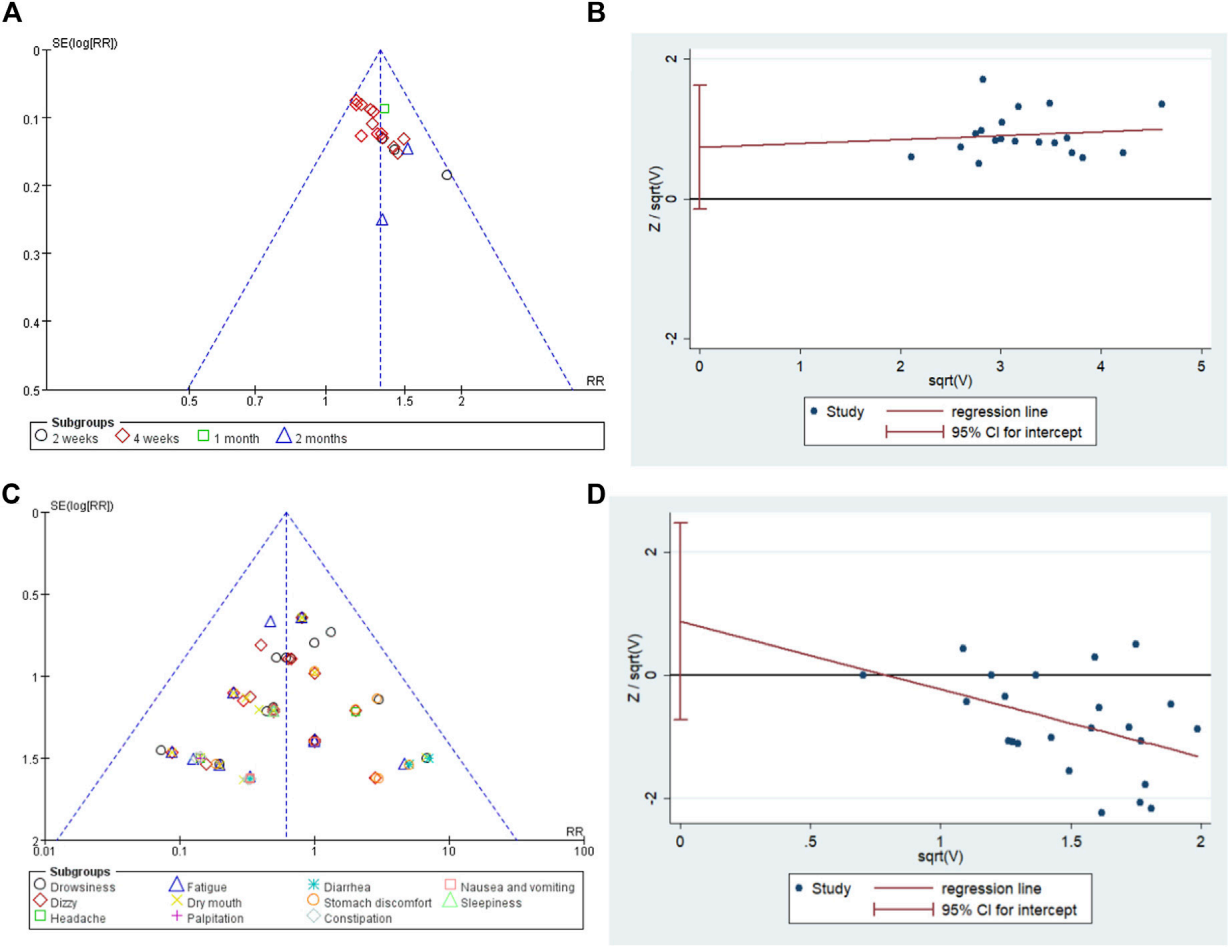
The results of publication bias. **(A)** Funnel plot of total effective rate, **(B)** Harbord tests plot of total effective rate, **(C)** funnel plot of the incidence of adverse events, **(D)** Harbord tests plot of the incidence of adverse events.

The meta-analyses on total effective rate and the incidence of adverse events included over ten studies. The publication bias of the two meta-analyses was tested. The funnel plot of the total effective rate and the total incidence of adverse events was not visually asymmetric. No significant publication biases were identified for total effective rate (T = 1.83, *p* = 0.084) and the incidence of adverse events (T = 1.14, *p* = 0.265) by Harbord tests. The details are shown in Figure 10.

## 4 Discussion

### 4.1 Summary of findings

In this study, 27 RCTs evaluating the efficacy of RZC combined with sgAHs for CU were identified. The results of the meta-analyses suggested that RZC combined with sgAHs therapy significantly improved the total effective rate in relative to sgAHs alone, decreased the recurrence rate, improved the quality of life measured by DLQI and enhanced serum IFN-γ level as well as reduced serum total IgE level and serum IL-4 level. Concerning safety, all the reported adverse events were mild and well tolerated, exerting no influence on the treatment. All the symptoms could spontaneously disappear after drug withdrawal.

Subgroup analyses indicate that compared with the sgAHs alone, RZC plus sgAHs can significantly improve the total effective rate, the quality of life measured by DLQI and serum IFN-γ level, and reduce serum total IgE level and serum IL-4 level when RZC is used for 2 weeks, 4 weeks, 1 month or 2 months. In addition, it suggests that the effect of RZC may be rapid and relatively sustained. Subgroup analyses on the recurrence rate based on the course of treatment and follow-up period demonstrate that combined with sgAHs, RZC exhibits the advantage of reducing long-term recurrence such as that after 1 month follow-up period, while the treatment duration may have no influence on the long-term recurrence rate. Based on the results of meta-analyses, RZC can reduce the incidence of dizziness, fatigue, dry mouth, and constipation. The incidences of other adverse effects including drowsiness, headaches, palpitation, diarrhea, stomach discomfort, nausea and vomiting, and sleepiness, are not of significant difference between the two groups. The safety of RZC may be satisfied.

Sensitivity analysis of DLQI, serum IgE level, serum IL-4 level, and serum IFN-γ level suggest that the pooled results are robust but do not identify the source of heterogeneity. Significant heterogeneity may be associated with factors including fewer included studies, small sample sizes, and inconsistent measurement methods of laboratory test indicators. Further discussion is not performed due to the lack of available data.

### 4.2 Comparison with previous studies

Several systematic reviews and meta-analyses showed the efficacy and safety of Chinese herbal medicine in combination with conventional western medicine for CU. However, there were some limitations in the studies that have been published.

Firstly, the course of the disease greater than or equal to 6 weeks is one of the vital items for diagnosing CU. However, participants with the unclear course of illness were all included in most researches (Xiao et al., 2020; Zheng et al., 2022), resulting in a possibility of lacking consistency between the research object and the research topic. The recognized diagnostic criteria, and a well-defined course of the disease are vital parts of our study. Secondly, different from the previous studies (Ke et al., 2021; Lin, 2022), no quasi-randomized trials were considered to ensure the quality of the literature. Thirdly, the prevention of CU recurrence is the advantage of TCM combination therapy. Some factors including the follow-up time and the course of treatment have influence on the recurrence rate to a certain extent, which has not been highlighted in the previously published studies (Liu et al., 2018; Zheng et al., 2022). However, the above factors were still discussed in this review despite being limited by the number of included studies.

### 4.3 Interpretation

Based on the theory of TCM, yin-blood deficiency and hemopenia generating wind are the primary etiology and pathogenesis of urticaria (Li, 2016). Combined with sgAHs, RZC with the functions of nourishing blood and yin, dispelling wind and arresting itching can increase clinical efficacy to a certain extent. The sgAHs, the preferred treatment for urticaria in modern medicine, are safer than the first-generation agents, while the occurrence of adverse events cannot be ignored. According to subgroup analysis of adverse events, the incidence of dizziness, fatigue, dry mouth and constipation in the RZC plus sgAHs group was notably lower than that in the sgAHs alone group. Based on the TCM theory, *R. multiflora* (Thunb.) Moldenke [Polygonaceae; Polygoni multiflori radix] with the function of supplementing essence blood, *R. glutinosa* (Gaertn.) DC. [Orobanchaceae; Rehmanniae radix] with the function of nourishing yin and generating fluid, and *M. alba* L. [Moraceae; Mori folium] with the function of moistening are the main components of RZC, which are effective in ameliorating dizziness, weakness, dry mouth and constipation (Bian et al., 2018; Cheng, 2019; Tang and Xu, 2021).

At present, the pathogenic mechanisms of CU are unclear. MCs are the essential effector cells in the pathogenesis of CU and can be activated through auto-immunity and non-autoimmunity (Kabashima et al., 2018; Maurer et al., 2019). Auto-immunity, either “autoallergic” (type I, with IgE antibodies to self-antigens/allergens) or “autoimmune” (type IIb, with IgG and IgM autoantibodies to IgE or its high-affinity receptor (FcεRI)) can cause MCs activation and degranulation (Wedi and Traidl, 2021). The autoallergy is a type I, IgE-mediated hypersensitivity reaction against self-antigens (Bracken et al., 2019). Autoallergic CU is related to IgE antibodies directed to self-antigens including thyroid peroxidase, thyroglobulin, and IL-24. It is different from classical type I hypersensitivity and allergy, involving exogenous allergens. Type II autoimmunity is featured by the appearance of IgG autoantibodies activating MCs, especially the IgG-anti-FcɛRI and IgG-anti-IgE (Maronese et al., 2023). Positive autologous serum skin test, immunoassays for IgG autoantibodies, and basophil activation tests are the current gold standard for the diagnosis (Kolkhir et al., 2021). CU patients with IgG autoantibodies have been divided into the autoimmune type IIb endotype because type IIb hypersensitivity is featured by an antibody-dependent process where specific IgG antibodies bind to autoantigens to create pathogenic states. It is different from type IIa involving cytolytic destruction of targeted cells (Kolkhir et al., 2022). Not all CU patients can be strictly classified as type I or type IIb autoimmunity. Recently, a milieu of co-existing between two endotypes in the pathophysiology of CU has been confirmed (Maronese et al., 2023). Some non-autoimmunity mechanisms include physical agents, pseudoallergens, infection, the local inflammatory milieu, eosinophils, and different T cell subsets (Kolkhir et al., 2020; Metz et al., 2014). The complex pathogenesis of CU provides an explanation for the existence of cases with refractory to the antihistamine treatment. The combination of RZC and sgAHs therapy plays synergistic effects on the treatment of CU.

RZC is consisted of *R. multiflora* (Thunb.) Moldenke [Polygonaceae; Polygoni multiflori radix], *R. glutinosa* (Gaertn.) DC. [Orobanchaceae; Rehmanniae radix], *M. alba* L. [Moraceae; Mori folium], *S. flavescens* Aiton [Fabaceae; Sophorae flavescentis radix], *L. bulbifera* (Siebold & Zucc.) Wedd. [Urticaceae; Laportea herba], which exerts its therapeutic effects for CU on multiple targets and pathways (Wang et al., 2023). *Rehmannia glutinosa* (Gaertn.) DC. [Orobanchaceae; Rehmanniae radix] can effectively lower the release of histamine through reducing IgE production and inhabit MCs activation, playing an anti-inflammatory role by improving interleukin-2 (IL-2) function (Sung et al., 2011; Kang et al., 2012). *Reynoutria multiflora* (Thunb.) Moldenke [Polygonaceae; Polygoni multiflori radix] block MCs degranulation and inhibit histamine release by promoting adrenocortical function (Li Y. X. et al., 2017; Lin et al., 2015); meanwhile, it also exerts anti-inflammatory activity by reducing the levels of interleukin-(IL-4), IL-5, IL-13, and other cytokines (Lee et al., 2016). *Sophora flavescens* Aiton [Fabaceae; Sophorae flavescentis radix] suppresses the MC-mediated histamine release. In addition, it also significantly reduces the release of 5-hydroxytryptamine to relieve the pruritus (Yamaguchi-Miyamoto et al., 2003; He et al., 2015). *Morus alba* L. [Moraceae; Mori folium] extract could reduce the plasma levels of IgE and histamine of atopic dermatitis mice induced by the house dust mite (Lim et al., 2014). It also inhibits NF-κB-mediated inflammatory response (Park et al., 2013). The main chemical component of *L. bulbifera* (Siebold & Zucc.) Wedd. [Urticaceae; Laportea herba] is total coumarins, with a content of over 50% (Zhang et al., 2013). The study demonstrated that total coumarins improved the atopic dermatitis symptoms of rats through reducing the production of IL-4, thymic stromal lymphopoietin (TSLP), and IgE and suppressed the pruritus caused by TSLP, and histamine (Yu et al., 2021). *Laportea bulbifera* (Siebold & Zucc.) Wedd. [Urticaceae; Laportea herba] can increase the expression of IL-10 and transforming growth factor-(TGF-) β in dendritic cells and induce the production of CD4^+^CD25^+^Treg cells for anti-inflammatory and immunosuppressive effects (Luo et al., 2011). Therefore, RZC can not only inhibit mast cell activation and degranulation through the auto-immune mechanism but also play a therapeutic role via other non-histamine-dependent pathways, such as anti-inflammatory, and immunosuppression. This conforms to the findings that RZC plus sgAHs therapy can improve the total effective rate, reduce serum total IgE and IL-4 level, and elevate the serum IFN-γ level.

### 4.4 Advantages and limitation

This is the first systematic review and meta-analysis on evaluating the efficacy and safety of RZC for CU. In line with the guidelines of the Cochrane Collaboration, it aims to draw more comprehensive and objective conclusions. By adding secondary outcome indicators, such as the expressions of inflammatory cytokines of IL-4 and IFN-γ, the efficacy of RZC combined with sgAHs therapy for CU can be evaluated in a multi-dimensional and multi-level manner. However, several potential limitations need to be pointed out when interpreting the above results.

1) In general, the methodological quality of the contained articles was low. Only less than half of the literature mentioned random sequence generation methods, and none of the included study involved allocation concealment and blinding. 2) All the recruited patients were Chinese individuals, and all the pieces of literature were published in Chinese, which may generate ethnic and geographical biases. 3) Only English or Chinese was used to search the literature, which may cause linguistic deviations. 4) The pooled results of several outcome indicators, such as DLQI, IgE, IL-4 and IFN-γ, suggested relatively high heterogeneity among the included studies. However, possible sources were not successfully analyzed and identified, even though the sensitivity analysis results were reliable.

### 4.5 Implications for future researches

There are some insights on how to improve the future trials on this topic. Firstly, the purpose should be clearly described, such as evaluate the efficacy of RZC alone or combined with western medicine for CU. Secondly, placebo-controlled RCTs are recommended. It is beneficial for implementing the blind and controlling the placebo effect. Thirdly, sample size should be estimated according to the appropriate parameters. This contributes to avoiding the bias in efficacy estimation. Fourthly, long follow-up period needs to be set up to identify the possible advantage of RZC on reducing the recurrence of CU. Fifthly, a protocol should be registered on an international clinical trial registration platform or published on an international academic journal. Trials should be reported following the Consolidated Standards of Reporting Trials (CONSORT) statement, which is helpful to acquire more detailed information.

## 5 Conclusion

According to available evidence, we found that RZC plus sgAHs may have more advantages than sgAHs alone in the treatment of CU, especially in improving the total effective rate and the quality of life, decreasing the recurrence rate, enhancing serum IFN-γ level and reducing serum total IgE level and serum IL-4 level. All the reported adverse events were mild and controllable. However, given that some elements of the risk of bias analysis including allocation concealment, blinding are not reported adequately, future trials on this topic need to be conducted more rigorously to obtain high-quality evidence, such as using placebo and long follow-up period, reasonably estimating sample size, registering the protocol, and reporting results according to CONSORT statement.

## Data Availability

The original contributions presented in the study are included in the article/Supplementary Material, further inquiries can be directed to the corresponding authors.
