# Supplementary material for: Efficacy and safety of Runzao Zhiyang capsule for chronic urticaria: a systematic review and meta-analysis of randomized controlled trials
Source: Front Pharmacol. 2023 Aug 24;14:1200252. doi: 10.3389/fphar.2023.1200252 (PMC10491456; doi:10.3389/fphar.2023.1200252)
Supplement: Supplementary file 1 [file DataSheet1.PDF]

Table of contents

Supplementary Material S1. Details of the Runzao Zhiyang Capsule……………………………………………………………………………………………2
Supplementary Material S2. Key active ingredients in Runzao Zhiyang Capsule………………………………………………………………………………..4
Supplementary Material S3. PRISMA 2020 checklist…….8
Supplementary Material S4. Search strategies for databases……………………………………………………………………………………………….12
Supplementary Material S5. Summary of composition characteristics of preparations in all included original studies…………………………………………14

Supplementary material S1 Details of the Runzao Zhiyang Capsule

Formulation	Source	SFDA approval number	Compositions	Description	Extraction procedure	Dosage and administration	Actions	Indications	Storage	Quality control reported? (Y/N)	Chemical analysis reported? (Y/N)
Runzao Zhiyang Capsule	China National Pharmaceutical Group Corporation Tongjitang (Guizhou) Pharmaceutical Co., Ltd.	Z20025030	*Reynoutria multiflora* (Thunb.) Moldenke [Polygonaceae; Polygoni multiflori radix]	This product is in capsule form, the contents are brownish-yellow to tan granules and powders; odor:fragrant, taste:slightly bitter.	*Reynoutria multiflora* (Thunb.) Moldenke [Polygonaceae; Polygoni multiflori radix], *Rehmannia glutinosa* (Gaertn.) DC. [Orobanchaceae; Rehmanniae radix], *Morus alba* L. [Moraceae; Mori folium], *Sophora flavescens* Aiton [Fabaceae; Sophorae flavescentis radix]; *Laportea bulbifera* (Siebold & Zucc.) Wedd. [Urticaceae; *Laportea* herba] were decocted in water three times, 1 hour each time. The decoction	0.5g per capsule. For oral administration, 4 capsules per time, three times a day, 2 weeks for a course of treatment.	Tonifying blood and nourishing yin, dispelling wind and arresting itching, relaxing bowels.	It is used for skin itching caused by blood deficiency and wind dryness, acne and constipation.	Seal	Y-prepared according to Pharmacopedia of the People's Republic of China (2000) and National Medical Products Administration Standards:WS-10029(ZD-0029)-2002-2011Z	Y-HPLC [Detail information can be got from Pharmacopoeia of the People's Republic of China (2000) (Part I, Appendix VI D)
			Rehmannia glutinosa (Gaertn.) DC. [Orobanchaceae; Rehmanniae radix]								
			Morus alba L. [Moraceae; Mori folium]								
			Sophora flavescens Aiton [Fabaceae; Sophorae flavescentis radix]								
			Laportea bulbifera (Siebold & Zucc.) Wedd. [Urticaceae; Laportea herba]								

					was filtered, and the filtrate was concentrated to a thick paste with a relative density of 1.38~1.42 (25℃), added with the fine powder of *Reynoutria multiflora* (Thunb.) Moldenke [Polygonaceae; Polygoni multiflori radix praeparata] above, mixed, dried at 75~80℃, pulverized, and put into capsules.						

The active ingredients of Runzao Zhiyang capsule were obtained using the traditional Chinese medicine systems pharmacology database and analysis platform (TCMSP) (https://tcmsp-e.com/) (screening conditions: the bioavailability (OB) ≥ 30% and drug-like properties (DL) ≥ 0.18). The data of *Morus alba* L. [Moraceae; Mori folium], *Sophora flavescens* Aiton [Fabaceae; Sophorae flavescentis radix] were obtained from TCMSP. Partial active ingredients of *Reynoutria multiflora* (Thunb.) Moldenke [Polygonaceae; Polygoni multiflori radix] and *Rehmannia glutinosa* (Gaertn.) DC. [Orobanchaceae; Rehmanniae radix] were got from the ETCM database (https://tcmip.cn/). The active ingredients of *Laportea bulbifera* (Siebold & Zucc.) Wedd. [Urticaceae; Laportea herba] were supplemented by the TCMSP and related article report. The results are as follows.

Supplementary Material S2 Key active ingredients in Runzao Zhiyang Capsule

**Compositions of Runzao Zhiyang Capsule**	**Chemicals**	**Resource**
Reynoutria multiflora (Thunb.) Moldenke [Polygonaceae; Polygoni multiflori radix]	Rhein	ETCM
	Moupinamide	ETCM
Rehmannia glutinosa (Gaertn.) DC. [Orobanchaceae; Rehmanniae radix]	beta-sitosterol	ETCM
	campesterol	ETCM
	coniferin	ETCM
	daucosterol	ETCM
Morus alba L. [Moraceae; Mori folium]	poriferast-5-en-3beta-ol	TCMSP
	beta-carotene	TCMSP
	Albanol	TCMSP
	Inophyllum E	TCMSP
	26-Hydroxy-dammara-20,24-dien-3-one	TCMSP
	Isoramanone	TCMSP
	Moracin B	TCMSP
	Moracin C	TCMSP
	Moracin D	TCMSP
	Moracin E	TCMSP
	Moracin F	TCMSP
	Moracin G	TCMSP

	Moracin H	TCMSP
	4-Prenylresveratrol	TCMSP
	Oxysanguinarine	TCMSP
	FA	TCMSP
	Oxysanguinarine	TCMSP
	quercetin	TCMSP
	beta-sitosterol	TCMSP
	kaempferol	TCMSP
	Stigmasterol	TCMSP
	arachidonic acid	TCMSP
	Supraene	TCMSP
	Iristectorigenin A	TCMSP
	icosa-11,14,17-trienoic acid methyl ester	TCMSP
	Norartocarpetin	TCMSP
	Linolenic acid ethyl ester	TCMSP
	Tetramethoxyluteolin	TCMSP
	Skimmin (8CI)	TCMSP
Sophora flavescens Aiton [Fabaceae; Sophorae flavescentis radix]	(2R)-5,7-dihydroxy-2-(4-hydroxyphenyl)chroman-4-one	TCMSP
	Inermine	TCMSP
	8-Isopentenyl-kaempferol	TCMSP
	sophocarpine	TCMSP
	Inermin	TCMSP
	Wighteone	TCMSP
	Sophoramine	TCMSP
	sophoridine	TCMSP
	formononetin	TCMSP

cis-Dihydroquercetin	TCMSP
(2R)-7-hydroxy-2-(4-hydroxyphenyl)chroman-4-one	TCMSP
5,7-dihydroxy-2-(3-hydroxy-4-methoxyphenyl)chroman-4-one	TCMSP
matrine	TCMSP
luteolin	TCMSP
(+)-14alpha-hydroxymatrine	TCMSP
(+)-7,11-dehydromatrine,(leontalbinine)	TCMSP
(+)-9alpha-hydroxymatrine	TCMSP
(+)-allomatrine	TCMSP
AIDS211310	TCMSP
(+)-lehmannine	TCMSP
isosophocarpine	TCMSP
(-)-14beta-hydroxymatrine	TCMSP
(-)-9alpha-hydroxysophoramine	TCMSP
anagyrine	TCMSP
1,4-diazaindan-type,alkaloid,flavascensine	TCMSP
13,14-dehydrosophoridine	TCMSP
5α,9α-dihydroxymatrine	TCMSP
7,11-dehydromatrine	TCMSP
Glyceollin	TCMSP
hyperforin	TCMSP
(2S)-7-hydroxy-2-(4-hydroxyphenyl)-5-methoxy-8-(3-methylbut-2-enyl)chroman-4-one	TCMSP
kushenin	TCMSP
kushenol J	TCMSP

	kushenol J_qt	TCMSP
	kushenol O	TCMSP
	kushenol,t	TCMSP
	leachianone,g	TCMSP
	Lehmanine	TCMSP
	(+)-Lupanine	TCMSP
	Norartocarpetin	TCMSP
	Phaseolin	TCMSP
	sophranol	TCMSP
	(-)-Maackiain-3-O-glucosyl-6'-O-malonate	TCMSP
	trifolrhizin	TCMSP
	quercetin	TCMSP
Laportea bulbifera (Siebold & Zucc.) Wedd. [Urticaceae; Laportea herba]	acacetin	Research article
	luteolin	Research article
	nobiletin	Research article
	(+)-catechin	Research article
	DNOP	Research article
	Ethyl linoleate	Research article
	Ethyl oleate	Research article

Note: DL: drug-like properties; OB: bioavailability; ETCM: ETCM database (https://tcmip.cn/); TCMSP: The traditional Chinese medicine systems pharmacology database and analysis platform (TCMSP) (https://tcmsp-e.com/).

Reference

Sun, K.F., Xu, W.F., Wang, B, Sun, Q.W., Dai, D.D. (2023). Research progress on the herbal research, chemical composition, pharmacological action and quality markers of Laportea herba. Chinese traditional Patent Medicine. 45(04):1222-1230.

Supplementary Material S3. PRISMA 2020 checklist

Section and Topic	Item #	Checklist item	Location where item is reported
**TITLE**			
Title	1	Identify the report as a systematic review.	P1
**ABSTRACT**			
Abstract	2	See the PRISMA 2020 for Abstracts checklist.	P1-2
**INTRODUCTION**			
Rationale	3	Describe the rationale for the review in the context of existing knowledge.	P2-3
Objectives	4	Provide an explicit statement of the objective(s) or question(s) the review addresses.	P2-3
**METHODS**			
Eligibility criteria	5	Specify the inclusion and exclusion criteria for the review and how studies were grouped for the syntheses.	P3-4
Information sources	6	Specify all databases, registers, websites, organisations, reference lists and other sources searched or consulted to identify studies. Specify the date when each source was last searched or consulted.	P4-5
Search strategy	7	Present the full search strategies for all databases, registers and websites, including any filters and limits used.	P4-5
Selection process	8	Specify the methods used to decide whether a study met the inclusion criteria of the review, including how many reviewers screened each record and each report retrieved, whether they worked independently, and if applicable, details of automation tools used in the process.	P5
Data collection process	9	Specify the methods used to collect data from reports, including how many reviewers collected data from each report, whether they worked independently, any processes for obtaining or confirming data from study investigators, and if applicable, details of automation tools used in the process.	P5
Data items	10a	List and define all outcomes for which data were sought. Specify whether all results that were compatible with each outcome domain in each study were sought (e.g. for all measures, time points, analyses), and if not, the methods used to decide which results to collect.	P5

Section and Topic	Item #	Checklist item	Location where item is reported
	10b	List and define all other variables for which data were sought (e.g. participant and intervention characteristics, funding sources). Describe any assumptions made about any missing or unclear information.	P5
Study risk of bias assessment	11	Specify the methods used to assess risk of bias in the included studies, including details of the tool(s) used, how many reviewers assessed each study and whether they worked independently, and if applicable, details of automation tools used in the process.	P5
Effect measures	12	Specify for each outcome the effect measure(s) (e.g. risk ratio, mean difference) used in the synthesis or presentation of results.	P5
Synthesis methods	13a	Describe the processes used to decide which studies were eligible for each synthesis (e.g. tabulating the study intervention characteristics and comparing against the planned groups for each synthesis (item #5)).	P5
	13b	Describe any methods required to prepare the data for presentation or synthesis, such as handling of missing summary statistics, or data conversions.	P5
	13c	Describe any methods used to tabulate or visually display results of individual studies and syntheses.	P5
	13d	Describe any methods used to synthesize results and provide a rationale for the choice(s). If meta-analysis was performed, describe the model(s), method(s) to identify the presence and extent of statistical heterogeneity, and software package(s) used.	P5
	13e	Describe any methods used to explore possible causes of heterogeneity among study results (e.g. subgroup analysis, meta-regression).	P5
	13f	Describe any sensitivity analyses conducted to assess robustness of the synthesized results.	P5
Reporting bias assessment	14	Describe any methods used to assess risk of bias due to missing results in a synthesis (arising from reporting biases).	P5
Certainty assessment	15	Describe any methods used to assess certainty (or confidence) in the body of evidence for an outcome.	P5
**RESULTS**			

Section and Topic	Item #	Checklist item	Location where item is reported
Study selection	16a	Describe the results of the search and selection process, from the number of records identified in the search to the number of studies included in the review, ideally using a flow diagram.	P6
	16b	Cite studies that might appear to meet the inclusion criteria, but which were excluded, and explain why they were excluded.	P6
Study characteristics	17	Cite each included study and present its characteristics.	P6
Risk of bias in studies	18	Present assessments of risk of bias for each included study.	P6
Results of individual studies	19	For all outcomes, present, for each study: (a) summary statistics for each group (where appropriate) and (b) an effect estimate and its precision (e.g. confidence/credible interval), ideally using structured tables or plots.	P6-10
Results of syntheses	20a	For each synthesis, briefly summarise the characteristics and risk of bias among contributing studies.	P6-10
	20b	Present results of all statistical syntheses conducted. If meta-analysis was done, present for each the summary estimate and its precision (e.g. confidence/credible interval) and measures of statistical heterogeneity. If comparing groups, describe the direction of the effect.	P6-10
	20c	Present results of all investigations of possible causes of heterogeneity among study results.	P6-10
	20d	Present results of all sensitivity analyses conducted to assess the robustness of the synthesized results.	P10
Reporting biases	21	Present assessments of risk of bias due to missing results (arising from reporting biases) for each synthesis assessed.	P10
Certainty of evidence	22	Present assessments of certainty (or confidence) in the body of evidence for each outcome assessed.	None
**DISCUSSION**			
Discussion	23a	Provide a general interpretation of the results in the context of other evidence.	P10-11

Section and Topic	Item #	Checklist item	Location where item is reported
	23b	Discuss any limitations of the evidence included in the review.	P11-13
	23c	Discuss any limitations of the review processes used.	P11-13
	23d	Discuss implications of the results for practice, policy, and future research.	P13-14
**OTHER INFORMATION**			
Registration and protocol	24a	Provide registration information for the review, including register name and registration number, or state that the review was not registered.	P3
	24b	Indicate where the review protocol can be accessed, or state that a protocol was not prepared.	P3
	24c	Describe and explain any amendments to information provided at registration or in the protocol.	P3
Support	25	Describe sources of financial or non-financial support for the review, and the role of the funders or sponsors in the review.	P14
Competing interests	26	Declare any competing interests of review authors.	P14-15
Availability of data, code and other materials	27	Report which of the following are publicly available and where they can be found: template data collection forms; data extracted from included studies; data used for all analyses; analytic code; any other materials used in the review.	P14

From: Page MJ, McKenzie JE, Bossuyt PM, Boutron I, Hoffmann TC, Mulrow CD, et al. The PRISMA 2020 statement: an updated guideline for reporting systematic reviews. BMJ 2021;372:n71. doi: 10.1136/bmj.n71

For more information, visit: http://www.prisma-statement.org/

Supplementary Material S4. Search strategies for databases

**PubMed**	#1 " Chronic Urticaria " [MeSH Terms] OR " Chronic Urticarias" [Title/Abstract] OR "Urticaria, Chronic" [Title/Abstract] OR "Chronic Spontaneous Urticaria" [Title/Abstract] OR "Chronic Spontaneous Urticarias" [Title/Abstract] OR "Spontaneous Urticaria, Chronic" [Title/Abstract] OR "Urticaria, Chronic Spontaneous" [Title/Abstract] OR "Idiopathic Chronic Urticaria" [Title/Abstract] OR "Chronic Urticaria, Idiopathic" [Title/Abstract] OR "Idiopathic Chronic Urticarias" [Title/Abstract] OR "Urticaria, Idiopathic Chronic" [Title/Abstract] OR "Chronic Idiopathic Urticaria" [Title/Abstract] OR "Chronic Idiopathic Urticarias" [Title/Abstract] OR "Idiopathic Urticaria, Chronic" [Title/Abstract] OR "Urticaria, Chronic Idiopathic" [Title/Abstract] OR "Chronic Autoimmune Urticaria" [Title/Abstract] OR "Autoimmune Urticaria, Chronic" [Title/Abstract] OR "Chronic Autoimmune Urticarias" [Title/Abstract] OR "Urticaria, Chronic Autoimmune" [Title/Abstract] OR "Autoimmune Urticaria" [Title/Abstract] OR "Autoimmune Urticarias" [Title/Abstract] OR "Urticaria, Autoimmune" [Title/Abstract]<br><br>#2 "Runzaozhiyang" [Title/Abstract] OR"Runzao zhiyang" [Title/Abstract] OR "Run Zao Zhi Yang" [Title/Abstract]<br><br>#3 "randomized controlled trial"[Title/Abstract] OR "controlled clinical trial"[Title/Abstract] OR" randomized " [Title/Abstract] OR" placebo" [Title/Abstract] OR" randomly " [Title/Abstract] OR" trial" [Title/Abstract]<br><br>#4 #1 AND #2 AND #3
**Web of Science**	#1 TS=(Chronic urticaria) OR TS=(Chronic urticarias) OR TS=(Chronic spontaneous urticaria) OR TS=(Chronic spontaneous urticarias) OR TS=(Idiopathic chronic urticaria) OR TS=(Idiopathic chronic urticaria) OR TS=(Chronic idiopathic urticaria) OR TS=(Chronic idiopathic urticarias) OR TS=(Chronic autoimmune urticaria) OR TS=(Chronic autoimmune urticarias) OR TS=(Autoimmune urticaria) OR TS=(Autoimmune urticarias)<br><br>#2 TS=(Runzaozhiyang) OR TS=(Runzao zhiyang) OR TS=(Run Zao Zhi Yang)<br><br>#3 TS=(randomized controlled trial) OR TS=(controlled clinical trial) OR TS=(randomized) OR TS=(placebo) OR TS=(randomly) OR TS=(trial)<br><br>#4 #1 AND #2 AND #3
**Embase**	#1 'chronic urticaria'/exp<br><br>#2 'chronic urticaria':ti,ab,kw OR 'chronic spontaneous urticaria':ti,ab,kw OR 'idiopathic chronic urticaria':ti,ab,kw OR 'chronic idiopathic urticaria':ti,ab,kw OR 'chronic autoimmune urticaria':ti,ab,kw OR 'autoimmune urticaria':ti,ab,kw<br><br>#3 #1 OR #2<br><br>#4 'runzaozhiyang':ti,ab,kw OR 'runzao zhiyang':ti,ab,kw OR 'run zao zhi yang':ti,ab,kw<br><br>#5 'randomized controlled trial':ab,ti,kw OR 'controlled clinical trial':ab,ti,kw OR 'randomized':ab,ti,kw OR 'placebo':ab,ti,kw OR 'randomly':ab,ti,kw OR 'trial':ab,ti,kw<br><br>#6 #3 AND #4 AND #5
**the Cochrane library**	#1 Title Abstract Keyword= (Chronic urticaria OR Chronic urticarias OR Chronic spontaneous urticaria OR Chronic spontaneous urticarias OR Idiopathic chronic urticaria OR Idiopathic chronic urticaria OR Chronic idiopathic urticaria OR Chronic idiopathic urticarias OR Chronic autoimmune urticaria OR Chronic autoimmune urticarias OR Autoimmune urticaria OR Autoimmune urticarias)

	#2 Title Abstract Keyword= (Runzaozhiyang OR Runzao Zhiyang OR Run zao zhi yang)<br><br>#3 Title Abstract Keyword= (randomized controlled trial OR controlled clinical trial OR randomized OR placebo OR randomly OR trial)<br><br>#4 #1 AND #2 AND #3
**China National Knowledge Infrastructure (CNKI)**	(SU=荨麻疹 OR SU=慢性荨麻疹 OR SU=风疹 OR SU=瘾疹) AND (SU=润燥止痒 OR SU=润燥止痒胶囊)
**Wanfang Database**	(主题词:荨麻疹 OR 主题词:慢性荨麻疹 OR 主题词: 风疹 OR 主题词:瘾疹) AND (主题词:润燥止痒 OR 主题词:润燥止痒胶囊)
**China Science and Technology Journal (VIP) Database**	U=(荨麻疹 OR 慢性荨麻疹 OR 风疹 OR 瘾疹) AND U=(润燥止痒 OR 润燥止痒胶囊)
**Chinese Biomedical Literature Database (SinoMed)**	("润燥止痒"[常用字段:智能] OR "润燥止痒胶囊"[常用字段:智能]) AND ("荨麻疹"[常用字段:智能] OR "慢性荨麻疹"[常用字段:智能] OR "风疹"[常用字段:智能] OR "瘾疹"[常用字段:智能])

Supplementary material S5 Summary of composition characteristics of preparations in all included original studies

Study	Formulation	Source	Species, concentration	Quality control reported? (Y/N)	Chemical analysis reported? (Y/N)
Yang (2019)	Runzao Zhiyang Capsule	China National Pharmaceutical Group Corporation Tongjitang (Guizhou) Pharmaceutical Co., Ltd.	*Reynoutria multiflora* (Thunb.) Moldenke [Polygonaceae; Polygoni multiflori radix]；*Rehmannia glutinosa* (Gaertn.) DC. [Orobanchaceae; Rehmanniae radix]；*Morus alba* L. [Moraceae; Mori folium]; *Sophora flavescens* Aiton [Fabaceae; Sophorae flavescentis radix]；*Laportea bulbifera* (Siebold & Zucc.) Wedd. [Urticaceae; Laportea herba] Concentration uncertainty of all ingredients; 0.5g/capsule	Y-prepared according to National Medical Products Administration Standard:WS-10029(ZD-0029)-2002-2011Z	N
Chen et al. (2016)	Runzao Zhiyang Capsule	China National Pharmaceutical Group Corporation Tongjitang (Guizhou) Pharmaceutical Co., Ltd.	*Reynoutria multiflora* (Thunb.) Moldenke [Polygonaceae; Polygoni multiflori radix]; *Rehmannia glutinosa* (Gaertn.) DC. [Orobanchaceae; Rehmanniae radix]；*Morus alba* L. [Moraceae; Mori folium]; *Sophora flavescens* Aiton [Fabaceae; Sophorae flavescentis radix]; *Laportea bulbifera* (Siebold & Zucc.) Wedd. [Urticaceae; Laportea herba] Concentration uncertainty of all ingredients; 0.5g/capsule	Y-prepared according to National Medical Products Administration Standard:WS-10029(ZD-0029)-2002-2011Z	N
Feng et al. (2011)	Runzao	China National Pharmaceutical	*Reynoutria multiflora* (Thunb.) Moldenke	Y-prepared according to National	Y-The effective component of

	Zhiyang Capsule	Group Corporation Tongjitang (Guizhou) Pharmaceutical Co., Ltd.	[Polygonaceae; Polygoni multiflori radix]; *Rehmannia glutinosa* (Gaertn.) DC. [Orobanchaceae; Rehmanniae radix]；*Morus alba* L. [Moraceae; Mori folium]; *Sophora flavescens* Aiton [Fabaceae; Sophorae flavescentis radix]; *Laportea bulbifera* (Siebold & Zucc.) Wedd. [Urticaceae; Laportea herba] Concentration uncertainty of all ingredients; 0.5g/capsule	Medical Products Administration Standard:WS-10029(ZD-0029)-2002-2011Z	*Rehmannia glutinosa* (Gaertn.) DC. [*Orobanchaceae;* Rehmanniae radix] are iridoid glycosides, which have anti-inflammatory, immune regulation and sedative effects; *Reynoutria multiflora* (Thunb.) Moldenke [Polygonaceae; Polygoni multiflori radix] contains anthraquinone compounds, which can promote the function of adrenal cortex and regulate the immune function.
Lv (2018)	Runzao Zhiyang Capsule	China National Pharmaceutical Group Corporation Tongjitang (Guizhou) Pharmaceutical Co., Ltd.	*Reynoutria multiflora* (Thunb.) Moldenke [Polygonaceae; Polygoni multiflori radix]; *Rehmannia glutinosa* (Gaertn.) DC. [Orobanchaceae; Rehmanniae radix]；*Morus alba* L. [Moraceae; Mori folium]; *Sophora flavescens* Aiton [Fabaceae; Sophorae flavescentis radix]; *Laportea bulbifera* (Siebold & Zucc.) Wedd. [Urticaceae; Laportea herba] Concentration uncertainty of all ingredients; 0.5g/capsule	Y-prepared according to National Medical Products Administration Standard:WS-10029(ZD-0029)-2002-2011Z	N
Luo et al. (2016)	Runzao Zhiyang Capsule	China National Pharmaceutical Group Corporation Tongjitang (Guizhou) Pharmaceutical Co., Ltd.	*Reynoutria multiflora* (Thunb.) Moldenke [Polygonaceae; Polygoni multiflori radix]; *Rehmannia glutinosa* (Gaertn.) DC.	N	N

			[Orobanchaceae; Rehmanniae radix] ; *Morus alba* L. [Moraceae; Mori folium]; *Sophora flavescens* Aiton [Fabaceae; Sophorae flavescentis radix]; *Laportea bulbifera* (Siebold & Zucc.) Wedd. [Urticaceae; Laportea herba]<br>Concentration uncertainty of all ingredients; 0.5g/capsule		
Tan (2018)	Runzao Zhiyang Capsule	China National Pharmaceutical Group Corporation Tongjitang (Guizhou) Pharmaceutical Co., Ltd.	*Reynoutria multiflora* (Thunb.) Moldenke [Polygonaceae; Polygoni multiflori radix]; *Rehmannia glutinosa* (Gaertn.) DC. [Orobanchaceae; Rehmanniae radix] ; *Morus alba* L. [Moraceae; Mori folium]; *Sophora flavescens* Aiton [Fabaceae; Sophorae flavescentis radix]; *Laportea bulbifera* (Siebold & Zucc.) Wedd. [Urticaceae; Laportea herba]<br>Concentration uncertainty of all ingredients; 0.5g/capsule	Y-prepared according to National Medical Products Administration Standard:WS-10029(ZD-0029)-2002-2011Z	N
Xiao (2020)	Runzao Zhiyang Capsule	China National Pharmaceutical Group Corporation Tongjitang (Guizhou) Pharmaceutical Co., Ltd.	*Reynoutria multiflora* (Thunb.) Moldenke [Polygonaceae; Polygoni multiflori radix]; *Rehmannia glutinosa* (Gaertn.) DC. [Orobanchaceae; Rehmanniae radix] ; *Morus alba* L. [Moraceae; Mori folium]; *Sophora flavescens* Aiton [Fabaceae; Sophorae flavescentis radix]; *Laportea*	Y-prepared according to National Medical Products Administration Standard:WS-10029(ZD-0029)-2002-2011Z	N

			bulbifera (Siebold & Zucc.) Wedd. [Urticaceae; Laportea herba]<br>Concentration uncertainty of all ingredients; 0.5g/capsule		
Li (2019)	Runzao Zhiyang Capsule	China National Pharmaceutical Group Corporation Tongjitang (Guizhou) Pharmaceutical Co., Ltd.	*Reynoutria multiflora* (Thunb.) Moldenke [Polygonaceae; Polygoni multiflori radix]; *Rehmannia glutinosa* (Gaertn.) DC. [Orobanchaceae; Rehmanniae radix] ; *Morus alba* L. [Moraceae; Mori folium]; *Sophora flavescens* Aiton [Fabaceae; Sophorae flavescentis radix]; *Laportea bulbifera* (Siebold & Zucc.) Wedd. [Urticaceae; Laportea herba]<br>Concentration uncertainty of all ingredients; 0.5g/capsule	Y-prepared according to National Medical Products Administration Standard:WS-10029(ZD-0029)-2002-2011Z	N
Feng et al. (2016)	Runzao Zhiyang Capsule	China National Pharmaceutical Group Corporation Tongjitang (Guizhou) Pharmaceutical Co., Ltd.	*Reynoutria multiflora* (Thunb.) Moldenke [Polygonaceae; Polygoni multiflori radix]; *Rehmannia glutinosa* (Gaertn.) DC. [Orobanchaceae; Rehmanniae radix] ; *Morus alba* L. [Moraceae; Mori folium]; *Sophora flavescens* Aiton [Fabaceae; Sophorae flavescentis radix]; *Laportea bulbifera* (Siebold & Zucc.) Wedd. [Urticaceae; Laportea herba]<br>Concentration uncertainty of all ingredients; 0.5g/capsule	Y-prepared according to National Medical Products Administration Standard:WS-10029(ZD-0029)-2002-2011Z	Y-*Sophora flavescens* Aiton [Fabaceae; Sophorae flavescentis radix] contains oxymatrine can reduce the release of allergic mediators, and prevent mast cells from degranulating and releasing histamine.

Liu and Li (2008)	Runzao Zhiyang Capsule	China National Pharmaceutical Group Corporation Tongjitang (Guizhou) Pharmaceutical Co., Ltd.	*Reynoutria multiflora* (Thunb.) Moldenke [Polygonaceae; Polygoni multiflori radix]; *Rehmannia glutinosa* (Gaertn.) DC. [Orobanchaceae; Rehmanniae radix] ； *Morus alba* L. [Moraceae; Mori folium]; *Sophora flavescens* Aiton [Fabaceae; Sophorae flavescentis radix]; *Laportea bulbifera* (Siebold & Zucc.) Wedd. [Urticaceae; Laportea herba] Concentration uncertainty of all ingredients; 0.5g/capsule	N	N
Li et al. (2017)	Runzao Zhiyang Capsule	China National Pharmaceutical Group Corporation Tongjitang (Guizhou) Pharmaceutical Co., Ltd.	*Reynoutria multiflora* (Thunb.) Moldenke [Polygonaceae; Polygoni multiflori radix]; *Rehmannia glutinosa* (Gaertn.) DC. [Orobanchaceae; Rehmanniae radix] ； *Morus alba* L. [Moraceae; Mori folium]; *Sophora flavescens* Aiton [Fabaceae; Sophorae flavescentis radix]; *Laportea bulbifera* (Siebold & Zucc.) Wedd. [Urticaceae; Laportea herba] Concentration uncertainty of all ingredients; 0.5g/capsule	Y-prepared according to National Medical Products Administration Standard:WS-10029(ZD-0029)-2002-2011Z	N
Tang and Xu (2021)	Runzao Zhiyang Capsule	China National Pharmaceutical Group Corporation Tongjitang (Guizhou) Pharmaceutical Co., Ltd.	*Reynoutria multiflora* (Thunb.) Moldenke [Polygonaceae; Polygoni multiflori radix]; *Rehmannia glutinosa* (Gaertn.) DC. [Orobanchaceae; Rehmanniae radix] ；	Y-prepared according to National Medical Products Administration Standard:WS-10029(ZD-0029)-2002-2011Z	N

			Morus alba L. [Moraceae; Mori folium]; *Sophora flavescens* Aiton [Fabaceae; Sophorae flavescentis radix]; *Laportea bulbifera* (Siebold & Zucc.) Wedd. [Urticaceae; Laportea herba]<br>Concentration uncertainty of all ingredients; 0.5g/capsule		
Bian et al. (2018)	Runzao Zhiyang Capsule	N	*Reynoutria multiflora* (Thunb.) Moldenke [Polygonaceae; Polygoni multiflori radix]; *Rehmannia glutinosa* (Gaertn.) DC. [Orobanchaceae; Rehmanniae radix]；*Morus alba* L. [Moraceae; Mori folium]; *Sophora flavescens* Aiton [Fabaceae; Sophorae flavescentis radix]; *Laportea bulbifera* (Siebold & Zucc.) Wedd. [Urticaceae; Laportea herba]<br>Concentration uncertainty of all ingredients; 0.5g/capsule	N	N
Du et al. (2018)	Runzao Zhiyang Capsule	China National Pharmaceutical Group Corporation Tongjitang (Guizhou) Pharmaceutical Co., Ltd.	*Reynoutria multiflora* (Thunb.) Moldenke [Polygonaceae; Polygoni multiflori radix]; *Rehmannia glutinosa* (Gaertn.) DC. [Orobanchaceae; Rehmanniae radix]；*Morus alba* L. [Moraceae; Mori folium]; *Sophora flavescens* Aiton [Fabaceae; Sophorae flavescentis radix]; *Laportea bulbifera* (Siebold & Zucc.) Wedd.	Y-prepared according to National Medical Products Administration Standard:WS-10029(ZD-0029)-2002-2011Z	N

			[Urticaceae; Laportea herba] Concentration uncertainty of all ingredients; 0.5g/capsule		
Liu and Yang (2019)	Runzao Zhiyang Capsule	China National Pharmaceutical Group Corporation Tongjitang (Guizhou) Pharmaceutical Co., Ltd.	*Reynoutria multiflora* (Thunb.) Moldenke [Polygonaceae; Polygoni multiflori radix]; *Rehmannia glutinosa* (Gaertn.) DC. [Orobanchaceae; Rehmanniae radix] ; *Morus alba* L. [Moraceae; Mori folium]; *Sophora flavescens* Aiton [Fabaceae; Sophorae flavescentis radix]; *Laportea bulbifera* (Siebold & Zucc.) Wedd. [Urticaceae; Laportea herba] Concentration uncertainty of all ingredients; 0.5g/capsule	N	N
Ai (2020)	Runzao Zhiyang Capsule	China National Pharmaceutical Group Corporation Tongjitang (Guizhou) Pharmaceutical Co., Ltd.	*Reynoutria multiflora* (Thunb.) Moldenke [Polygonaceae; Polygoni multiflori radix]; *Rehmannia glutinosa* (Gaertn.) DC. [Orobanchaceae; Rehmanniae radix] ; *Morus alba* L. [Moraceae; Mori folium]; *Sophora flavescens* Aiton [Fabaceae; Sophorae flavescentis radix]; *Laportea bulbifera* (Siebold & Zucc.) Wedd. [Urticaceae; Laportea herba] Concentration uncertainty of all ingredients; 0.5g/capsule	Y-prepared according to National Medical Products Administration Standard:WS-10029(ZD-0029)-2002-2011Z	N
Zhou (2020)	Runzao	China National Pharmaceutical	*Reynoutria multiflora* (Thunb.) Moldenke	Y-prepared according to National	N

	Zhiyang Capsule	Group Corporation Tongjitang (Guizhou) Pharmaceutical Co., Ltd.	[Polygonaceae; Polygoni multiflori radix]; *Rehmannia glutinosa* (Gaertn.) DC. [Orobanchaceae; Rehmanniae radix] ; *Morus alba* L. [Moraceae; Mori folium]; *Sophora flavescens* Aiton [Fabaceae; Sophorae flavescentis radix]; *Laportea bulbifera* (Siebold & Zucc.) Wedd. [Urticaceae; Laportea herba]<br>Concentration uncertainty of all ingredients; 0.5g/capsule	Medical Products Administration Standard:WS-10029(ZD-0029)-2002-2011Z	
Ma et al. (2014)	Runzao Zhiyang Capsule	China National Pharmaceutical Group Corporation Tongjitang (Guizhou) Pharmaceutical Co., Ltd.	*Reynoutria multiflora* (Thunb.) Moldenke [Polygonaceae; Polygoni multiflori radix]; *Rehmannia glutinosa* (Gaertn.) DC. [Orobanchaceae; Rehmanniae radix] ; *Morus alba* L. [Moraceae; Mori folium]; *Sophora flavescens* Aiton [Fabaceae; Sophorae flavescentis radix]; *Laportea bulbifera* (Siebold & Zucc.) Wedd. [Urticaceae; Laportea herba]<br>Concentration uncertainty of all ingredients; 0.5g/capsule	N	Y-*Sophora flavescens* Aiton [Fabaceae; Sophorae flavescentis radix] contains various alkaloids, which have anti-inflammatory, anti-allergic, and immunosuppressive effects.
Sun (2014)	Runzao Zhiyang Capsule	China National Pharmaceutical Group Corporation Tongjitang (Guizhou) Pharmaceutical Co., Ltd.	*Reynoutria multiflora* (Thunb.) Moldenke [Polygonaceae; Polygoni multiflori radix]; *Rehmannia glutinosa* (Gaertn.) DC. [Orobanchaceae; Rehmanniae radix] ; *Morus alba* L. [Moraceae; Mori folium];	Y-prepared according to National Medical Products Administration Standard:WS-10029(ZD-0029)-2002-2011Z	N

			Sophora flavescens Aiton [Fabaceae; Sophorae flavescentis radix]; *Laportea bulbifera* (Siebold & Zucc.) Wedd. [Urticaceae; Laportea herba]<br>Concentration uncertainty of all ingredients; 0.5g/capsule		
Chen et al. (2020)	Runzao Zhiyang Capsule	China National Pharmaceutical Group Corporation Tongjitang (Guizhou) Pharmaceutical Co., Ltd.	*Reynoutria multiflora* (Thunb.) Moldenke [Polygonaceae; Polygoni multiflori radix]; *Rehmannia glutinosa* (Gaertn.) DC. [Orobanchaceae; Rehmanniae radix]；*Morus alba* L. [Moraceae; Mori folium]; *Sophora flavescens* Aiton [Fabaceae; Sophorae flavescentis radix]; *Laportea bulbifera* (Siebold & Zucc.) Wedd. [Urticaceae; Laportea herba]<br>Concentration uncertainty of all ingredients; 0.5g/capsule	Y-prepared according to National Medical Products Administration Standard:WS-10029(ZD-0029)-2002-2011Z	Y-*Sophora flavescens* Aiton [Fabaceae; Sophorae flavescentis radix] contains oxymatrine can reduce the release of allergic mediators, and prevent mast cells from degranulating.
Zhang (2020)	Runzao Zhiyang Capsule	N	*Reynoutria multiflora* (Thunb.) Moldenke [Polygonaceae; Polygoni multiflori radix]; *Rehmannia glutinosa* (Gaertn.) DC. [Orobanchaceae; Rehmanniae radix]；*Morus alba* L. [Moraceae; Mori folium]; *Sophora flavescens* Aiton [Fabaceae; Sophorae flavescentis radix]; *Laportea bulbifera* (Siebold & Zucc.) Wedd. [Urticaceae; Laportea herba]	N	N

			Concentration uncertainty of all ingredients; 0.5g/capsule		
Wang and Fang (2013)	Runzao Zhiyang Capsule	China National Pharmaceutical Group Corporation Tongjitang (Guizhou) Pharmaceutical Co., Ltd.	*Reynoutria multiflora* (Thunb.) Moldenke [Polygonaceae; Polygoni multiflori radix]; *Rehmannia glutinosa* (Gaertn.) DC. [Orobanchaceae; Rehmanniae radix] ； *Morus alba* L. [Moraceae; Mori folium]; *Sophora flavescens* Aiton [Fabaceae; Sophorae flavescentis radix]; *Laportea bulbifera* (Siebold & Zucc.) Wedd. [Urticaceae; Laportea herba] Concentration uncertainty of all ingredients; 0.5g/capsule	Y-prepared according to National Medical Products Administration Standard:WS-10029(ZD-0029)-2002-2011Z	N
Cheng (2019)	Runzao Zhiyang Capsule	China National Pharmaceutical Group Corporation Tongjitang (Guizhou) Pharmaceutical Co., Ltd.	*Reynoutria multiflora* (Thunb.) Moldenke [Polygonaceae; Polygoni multiflori radix]; *Rehmannia glutinosa* (Gaertn.) DC. [Orobanchaceae; Rehmanniae radix] ； *Morus alba* L. [Moraceae; Mori folium]; *Sophora flavescens* Aiton [Fabaceae; Sophorae flavescentis radix]; *Laportea bulbifera* (Siebold & Zucc.) Wedd. [Urticaceae; Laportea herba] Concentration uncertainty of all ingredients; 0.5g/capsule	Y-prepared according to National Medical Products Administration Standard:WS-10029(ZD-0029)-2002-2011Z	N
Wang et al. (2018)	Runzao Zhiyang	China National Pharmaceutical Group Corporation Tongjitang	*Reynoutria multiflora* (Thunb.) Moldenke [Polygonaceae; Polygoni multiflori radix];	N	N

	Capsule	(Guizhou) Pharmaceutical Co., Ltd.	*Rehmannia glutinosa* (Gaertn.) DC. [Orobanchaceae; Rehmanniae radix]；*Morus alba* L. [Moraceae; Mori folium]; *Sophora flavescens* Aiton [Fabaceae; Sophorae flavescentis radix]; *Laportea bulbifera* (Siebold & Zucc.) Wedd. [Urticaceae; Laportea herba] Concentration uncertainty of all ingredients; 0.5g/capsule		
Zhang (2014)	Runzao Zhiyang Capsule	China National Pharmaceutical Group Corporation Tongjitang (Guizhou) Pharmaceutical Co., Ltd.	*Reynoutria multiflora* (Thunb.) Moldenke [Polygonaceae; Polygoni multiflori radix]; *Rehmannia glutinosa* (Gaertn.) DC. [Orobanchaceae; Rehmanniae radix]；*Morus alba* L. [Moraceae; Mori folium]; *Sophora flavescens* Aiton [Fabaceae; Sophorae flavescentis radix]; *Laportea bulbifera* (Siebold & Zucc.) Wedd. [Urticaceae; Laportea herba] Concentration uncertainty of all ingredients; 0.5g/capsule	Y-prepared according to National Medical Products Administration Standard:WS-10029(ZD-0029)-2002-2011Z	N
Huang (2011)	Runzao Zhiyang Capsule	China National Pharmaceutical Group Corporation Tongjitang (Guizhou) Pharmaceutical Co., Ltd.	*Reynoutria multiflora* (Thunb.) Moldenke [Polygonaceae; Polygoni multiflori radix]; *Rehmannia glutinosa* (Gaertn.) DC. [Orobanchaceae; Rehmanniae radix]；*Morus alba* L. [Moraceae; Mori folium]; *Sophora flavescens* Aiton [Fabaceae;	N	N

			Sophorae flavescentis radix]; *Laportea bulbifera* (Siebold & Zucc.) Wedd. [Urticaceae; Laportea herba]<br>Concentration uncertainty of all ingredients; 0.5g/capsule		
Zhang (2010)	Runzao Zhiyang Capsule	China National Pharmaceutical Group Corporation Tongjitang (Guizhou) Pharmaceutical Co., Ltd.	*Reynoutria multiflora* (Thunb.) Moldenke [Polygonaceae; Polygoni multiflori radix]; *Rehmannia glutinosa* (Gaertn.) DC. [Orobanchaceae; Rehmanniae radix] ; *Morus alba* L. [Moraceae; Mori folium]; *Sophora flavescens* Aiton [Fabaceae; Sophorae flavescentis radix]; *Laportea bulbifera* (Siebold & Zucc.) Wedd. [Urticaceae; Laportea herba]<br>Concentration uncertainty of all ingredients; 0.5g/capsule	N	N

Notes: Concentration of each species in the preparations belong to the core technology of the pharmaceutical companies, so they did not report the grams of each composition.
